# New WHO prevalence estimates of mental disorders in conflict settings: a systematic review and meta-analysis

**DOI:** 10.1016/S0140-6736(19)30934-1

**Published:** 2019-07-20

**Authors:** Fiona Charlson, Mark van Ommeren, Abraham Flaxman, Joseph Cornett, Harvey Whiteford, Shekhar Saxena

**Affiliations:** a Policy and Epidemiology Group, Queensland Centre for Mental Health Research, QLD, Australia; b School of Public Health, University of Queensland, QLD, Australia; c Institute for Health Metrics and Evaluation, University of Washington, Seattle, WA, USA; d Department of Mental Health and Substance Abuse, WHO, Geneva, Switzerland; e T H Chan School of Public Health, Harvard University, Boston, MA, USA

## Abstract

**Background:**

Existing WHO estimates of the prevalence of mental disorders in emergency settings are more than a decade old and do not reflect modern methods to gather existing data and derive estimates. We sought to update WHO estimates for the prevalence of mental disorders in conflict-affected settings and calculate the burden per 1000 population.

**Methods:**

In this systematic review and meta-analysis, we updated a previous systematic review by searching MEDLINE (PubMed), PsycINFO, and Embase for studies published between Jan 1, 2000, and Aug 9, 2017, on the prevalence of depression, anxiety disorder, post-traumatic stress disorder, bipolar disorder, and schizophrenia. We also searched the grey literature, such as government reports, conference proceedings, and dissertations, to source additional data, and we searched datasets from existing literature reviews of the global prevalence of depression and anxiety and reference lists from the studies that were identified. We applied the Guidelines for Accurate and Transparent Health Estimates Reporting and used Bayesian meta-regression techniques that adjust for predictors of mental disorders to calculate new point prevalence estimates with 95% uncertainty intervals (UIs) in settings that had experienced conflict less than 10 years previously.

**Findings:**

We estimated that the prevalence of mental disorders (depression, anxiety, post-traumatic stress disorder, bipolar disorder, and schizophrenia) was 22·1% (95% UI 18·8–25·7) at any point in time in the conflict-affected populations assessed. The mean comorbidity-adjusted, age-standardised point prevalence was 13·0% (95% UI 10·3–16·2) for mild forms of depression, anxiety, and post-traumatic stress disorder and 4·0% (95% UI 2·9–5·5) for moderate forms. The mean comorbidity-adjusted, age-standardised point prevalence for severe disorders (schizophrenia, bipolar disorder, severe depression, severe anxiety, and severe post-traumatic stress disorder) was 5·1% (95% UI 4·0–6·5). As only two studies provided epidemiological data for psychosis in conflict-affected populations, existing Global Burden of Disease Study estimates for schizophrenia and bipolar disorder were applied in these estimates for conflict-affected populations.

**Interpretation:**

The burden of mental disorders is high in conflict-affected populations. Given the large numbers of people in need and the humanitarian imperative to reduce suffering, there is an urgent need to implement scalable mental health interventions to address this burden.

**Funding:**

WHO; Queensland Department of Health, Australia; and Bill & Melinda Gates Foundation.

## Introduction

Currently, there are major conflict-induced humanitarian crises in numerous countries, including Afghanistan, Iraq, Nigeria, Somalia, South Sudan, Syria, and Yemen. UN estimates suggest that more than 68·6 million people worldwide have been forcibly displaced by violence and conflict, the highest number of people affected since World War 2.^1^ This increase in people affected by conflict coincides with a growing interest in mental health, as exemplified by the recently approved 10-year extension of the Mental Health Action Plan by 194 WHO member states.^2^ Interest is especially high in the mental health of people affected by humanitarian emergencies.^3^

In 2005, WHO estimated the prevalence of mental disorders among people affected by humanitarian emergencies.^4^ These estimates have been frequently repeated in policy documents,^3^, ^5^, ^6^ news media,^7^ and appeals and funding proposals for help for people living through the world's worst crises. WHO emphasised that these estimates represented averages across emergency settings and that observed prevalence estimates would vary by affected population and assessment method.^4^ However, WHO's 2005 estimates were not based on applicable systematic reviews of evidence.

Epidemiological studies in conflict settings typically present varying results, making their interpretation difficult,^8^ and their statistical heterogeneity is extremely high.^9^, ^10^ We sought to update WHO estimates of the prevalence of mental disorders in conflict-affected populations by updating systematic literature reviews for post-traumatic stress disorder and depression, searching for a wider range of disorders, and applying Bayesian meta-regression techniques while adjusting for predictors of mental disorders in conflict settings. Natural disasters and public health emergencies, such as the Ebola virus outbreak, were outside the scope of this study. Previous research has identified different mental health consequences across these emergency settings^11^, ^12^ (conflict probably has more severe consequences), and our selectivity was designed to limit heterogeneity within our dataset. Our approach was in line with current WHO and Inter-Agency Standing Committee humanitarian policies and tools that include a broad multidisorder perspective.^2^, ^3^, ^13^, ^14^ Furthermore, we aimed to estimate disease burden in terms of years lived with disability (YLDs) per 1000 people affected by conflict.

**Research in context**

**Evidence before this study**

In 2005, in response to the Asian tsunami, WHO estimated the prevalence of mental disorders among people affected by humanitarian emergencies. These estimates were repeated in policy documents, news media, and appeals and funding proposals, but they did not have confidence intervals and were not based on systematic reviews of evidence. We searched MEDLINE (PubMed), PsycINFO, and Embase, to identify studies published from Jan 1, 2000, to Aug 9, 2017, to identify sources for the prevalence of post-traumatic stress disorder, depression, and anxiety disorders using the Diagnostic and Statistical Manual of Mental Disorders (DSM) or the International Classification of Diseases (ICD) criteria and variables known to be associated with prevalence (such as exposure to trauma) to guide a predictor analysis. We used the search string ((((((((“Warfare”[MESH]) OR “Warfare and Armed Conflicts”[MESH]) OR “Torture”[MESH]) OR “Ethnic Violence”[MESH]) OR “Exposure to Violence”[MESH]) OR “Mass Casualty Incidents”[MESH]) OR “Civil Disorders”[MESH])) AND (((((“Anxiety Disorders”[MESH]) OR “Mood Disorders”[MESH]) OR “Trauma and Stressor Related Disorders”[MESH]) OR “Stress, Psychological”[MESH]) OR “Neurotic Disorders”[MESH]) AND ((((“Epidemiology”[MESH] OR “epidemiology” [Subheading]) OR “Prevalence”[MESH]) OR “Psychiatric Status Rating Scales”[MESH])) for PubMed, and adapted it for the other online databases. No language restriction was applied. We also did a grey literature search using Google Scholar, datasets from existing literature reviews, and reference lists from studies identified.

**Added value of this study**

In this systematic review and meta-analysis, we updated WHO's 2005 estimates for the prevalence of mental disorders in conflict-affected low-income and middle-income settings, focusing on depression, anxiety disorder, post-traumatic stress disorder, bipolar disorder, and schizophrenia in settings that had experienced conflict in the preceding 10 years. We estimated that more than one in five people (22·1%) in post-conflict settings has depression, anxiety disorder, post-traumatic stress disorder, bipolar disorder, or schizophrenia and that almost one in ten people (9·1%) in post-conflict settings has a moderate of severe mental disorder at any point in time.

**Implications of all the available evidence**

Given that the prevalence of mental disorders was found to be very high, there is a need to make available sustainable mental health care in conflict-affected countries. This will require a focus on investment in leadership and governance for mental health; integrated and responsive mental health and social care services in community-based settings; strategies for promotion and prevention in mental health; and information systems, evidence, and research for mental health in conflict-affected countries. The well established links between mental health, individual functioning, and country development underscore the imperative to prioritise mental health care in countries affected by conflict.

## Methods

### Overview

For this systematic review and meta-analysis, we followed the Guidelines for Accurate and Transparent Health Estimates Reporting (GATHER) statement^15^ and used methodologies developed for the Global Burden of Diseases, Injuries, and Risk Factors Study (GBD) 2016.^16^

We refer to the generic term conflict as a substitute for armed conflict and war. Current concepts and definitions of conflict were extracted by searching peace and conflict databases, organisation websites, and published reports. The relevance and usefulness of current concepts and definitions of conflict were assessed to determine the most appropriate database for our context. A critique of the usefulness of each database identified five potentially appropriate conflict databases. The Uppsala Conflict Data Program,^17^ the Correlates of War project,^18^ the Integrated Network for Societal Conflict Research Major Episodes of Political Violence,^19^ and the Heidelberg Institute for International Conflict Research Conflict Barometer^20^ describe conflict as the existence of opposing forces and stipulate a violence threshold described in terms of number of deaths. The Political Terror Scale^21^ reports level of state terror according to state-perpetrated human rights violations. We then did a quantitative assessment of concordance between these five databases using the kappa (κ) statistic.^22^ On the basis of the assessments of usefulness and concordance, we elected to use both the Uppsala Conflict Data Program and Political Terror Scale databases. Further details of this process can be found in the appendix and online.^23^

We based our dataset on a previous systematic review,^10^ which included studies published between 1980 and 2013 (search details in the appendix). We updated this review by searching MEDLINE (PubMed), PsycINFO, and Embase for studies published between Jan 1, 2000, and Aug 9, 2017, to identify sources for the prevalence of post-traumatic stress disorder, depression, anxiety disorders, schizophrenia, and bipolar disorder diagnosed using the Diagnostic and Statistical Manual of Mental Disorders (DSM) or the International Classification of Diseases (ICD) criteria, and for variables known to be associated with prevalence (such as exposure to trauma) to guide a predictor analysis. No language restriction was applied. We also searched the grey literature, such as government reports, conference proceedings, and dissertations, to source additional data. Sources included Google search engines (eg, Google Scholar) and ProQuest digital dissertations. Additionally, we searched datasets from existing literature reviews of the global prevalence of depression and anxiety^24^, ^25^ and reference lists from the studies that were identified. All grey-literature sources identified were in English. We sought data on the prevalence of schizophrenia and bipolar disorder in conflict-affected populations from existing systematic reviews.^26^, ^27^

We included study samples that were representative of the general conflict-affected population, defined as being within a described geographical location and having been in a state of conflict within 10 years preceding data collection, as documented by the Uppsala Conflict Data Program database.^17^ We only included studies of participants residing in their country of origin, or displaced or resettled in a neighbouring low-income or middle-income country; populations resettled in a high-income country were excluded because there is evidence that the heterogeneity might be considerable because of exposure to external and environmental factors during the resettling process.^9^ We included studies that reported point or past-year prevalence estimates from either cross-sectional or longitudinal population-based surveys. Survey instruments had to map to DSM or ICD diagnostic criteria. A complete list of inclusion and exclusion criteria can be found in the appendix.

Two of the authors (FC and JC) were responsible for the searches and data extraction. Identified data sources were reviewed by both authors and, if a consensus was not reached, a third author (HW).

### Data analysis

Data were extracted into a standardised excel template. Duplicate data from the same study samples and reported in multiple studies were identified and removed. We used a Bayesian meta-regression model and the adaptive Metropolis Markov-chain Monte Carlo method to draw samples from the posterior distribution of all model parameters simultaneously, with the modelling software package DisMod-MR 1.0.^16^, ^28^ To explain between-study variability in prevalence, we included a range of study-level and war-related covariates that had previously been shown to have significant associations with mental disorder prevalence.^10^ We reported age-standardised point estimates based on the means of functions of these parameter draws, and uncertainty intervals (UIs) corresponding to the 2·5–97·5 percentile values. The UI provides an upper and lower bound that the model predicts to contain the true value with 95% certainty. Details on covariate selection can be found in the appendix.

We conducted quality assessment at the time of data extraction through our inclusion and exclusion criteria.

To adjust for comorbidities and severity splits in depression, anxiety, and post-traumatic stress disorder, we applied the prevalence of 41·6% (95% UI 39·8–43·4) of individuals with depressive disorder who also had comorbid anxiety, as previously identified from the literature.^29^ Distributions of depression and anxiety severity were taken from GBD 2016,^16^ which considers several health states within a particular disease that are reflective of different levels of functional impairment (ie, none, mild, moderate, or severe) once disability attributable to comorbid disorders is portioned out.^30^ In the absence of severity splits for post-traumatic stress disorder, we relied on severity distributions for anxiety disorders. More detail on GBD severity splits and disability weights can be found in the appendix.

YLDs were derived by multiplying the number of prevalent cases associated with each disorder by their associated GBD disability weight. In place of GBD prevalence estimates, we used prevalence estimates of conflict-affected population mental disorder (derived as described previously) as a primary input for YLD estimation. Post-traumatic stress disorder was conceptualised as an anxiety disorder until the most recent version of DSM (fifth edition) and, accordingly, was not assessed as a separate disorder in GBD 2016, so we did not calculate burden of disease estimates for post-traumatic stress disorder. In our analyses, we considered all prevalent cases of schizophrenia and bipolar disorder as severe. We used Monte Carlo simulation–modelling techniques to present 95% UIs around estimates reflecting the main sources of sampling uncertainty in the calculations using Ersatz software, version 1.2.^31^ More detailed information on GBD burden of disease estimation can be found elsewhere.^16^, ^32^

### Role of the funding source

WHO provided funding for this study and had a role in study design, data interpretation, and writing of the report. The other funders had no role in study design, data collection, data analysis, data interpretation, or writing of the report. The corresponding author had full access to all the data in the study and had final responsibility for the decision to submit for publication.

## Results

**Figure 1:**
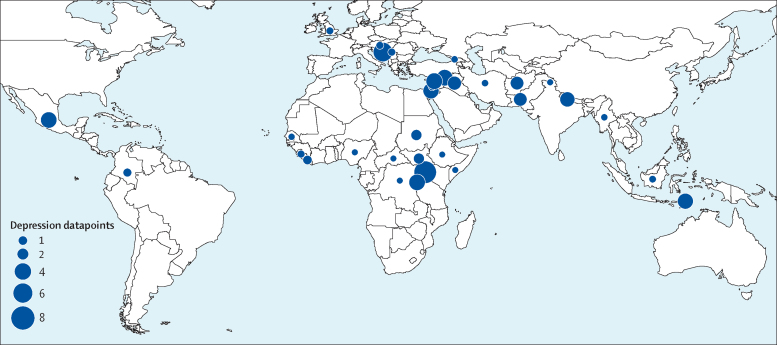
Map of number of depression studies, 1980–2017

**Figure 2:**
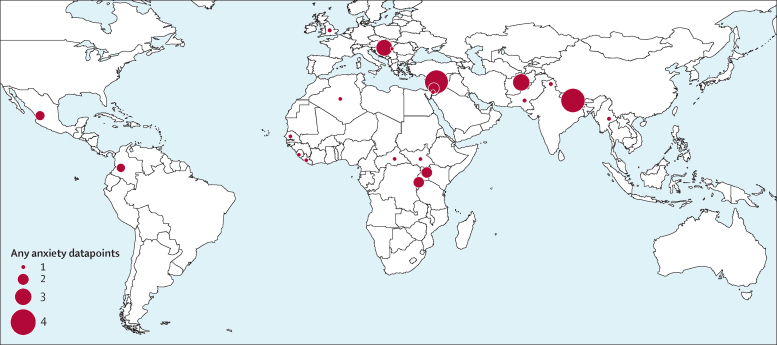
Map of number of any anxiety studies, 1980–2017

**Figure 3:**
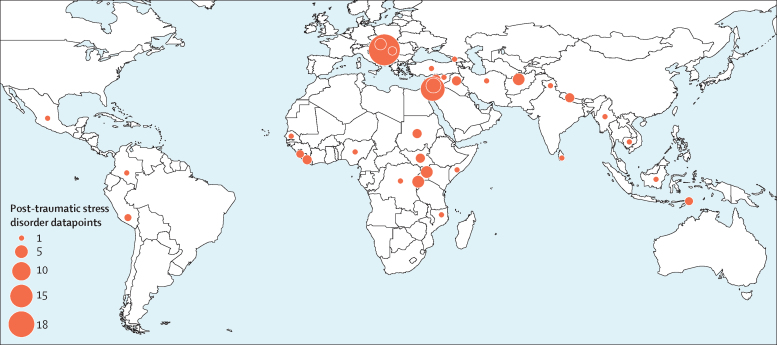
Map of number of post-traumatic stress disorder studies, 1980–2017

We identified 129 studies published between Jan 1, 1980, and Aug 9, 2017, providing 96 studies with prevalence estimates for post-traumatic stress disorder, 70 studies with prevalence estimates for depression, and 38 studies with prevalence estimates for any anxiety disorder (appendix); 51 of these were studies published between Jan 1, 2000, and Aug 9, 2017. 39 countries were represented in the dataset; 34 had data for depression, 34 had data for post-traumatic stress disorder, and 25 had data for anxiety (Figure 1, Figure 2, Figure 3; appendix).

We estimated that the prevalence of mental disorders (depression, anxiety, post-traumatic stress disorder, bipolar disorder, and schizophrenia) was 22·1% (95% UI 18·8–25·7) at any point in time in the conflict-affected populations assessed (table 1). Age-standardised prevalence for depression, post-traumatic stress disorder, and anxiety disorders was elevated in conflict-affected populations compared with global mean prevalence (10·8% [95% UI 8·1–14·2] for depression, 15·3% [9·9–23·5] for post-traumatic stress disorder, and 21·7% [16·7–28·3] for any anxiety disorders; table 2). The mild forms of all three disorders were the most prevalent. Adjusting for comorbidity between depression and anxiety, the mean, combined age-standardised prevalence of mild, moderate, and severe depression, post-traumatic stress disorder, and any anxiety disorders was 21·2% [95% UI 17·7–24·7] in conflict-affected populations (table 3). The mean comorbidity-adjusted, age-standardised point prevalence was 13·0% (95% UI 10·3–16·2) for mild forms of depression, anxiety, and post-traumatic stress disorder and 4·0% (95% UI 2·9–5·5) for moderate forms. The mean comorbidity-adjusted, age-standardised point prevalence for severe disorders (schizophrenia, bipolar disorder, severe depression, severe anxiety, and severe post-traumatic stress disorder) was 5·1% (95% UI 4·0–6·5). By aggregating the prevalence of mental disorders in conflict-affected populations by severity, we estimated that at any point in time about 9% of the conflict-affected population has moderate to severe mental disorders (schizophrenia, bipolar disorder, moderate to severe anxiety, moderate to severe post-traumatic stress disorder, and moderate to severe depression; table 1).

**Table 1:** Point prevalence estimates for mental disorders in conflict-affected populations, adjusted for comorbidity

	**Point prevalence (95% uncertainty interval)**
Severe disorder (severe anxiety, severe post-traumatic stress disorder, severe depression, schizophrenia, and bipolar disorder)	5·1% (4·0–6·5)
Moderate disorder (moderate anxiety, moderate post-traumatic stress disorder, and moderate depression)	4·0% (2·9–5·5)
Mild disorder (mild anxiety, mild post-traumatic stress disorder, and mild depression)	13·0% (10·3–16·2)
Total	22·1% (18·8–25·7)

**Table 2:** Age-standardised point prevalence with 95% uncertainty intervals, unadjusted for comorbidity

	**Depression**	**Any anxiety disorder (including post-traumatic stress disorder)**	**Post-traumatic stress disorder**
Severe disorder	1·1% (0·3–2·2)	2·8% (1·8–4·0)	2·0% (1·1–3·2)
Moderate disorder	1·8% (1·2–2·6)	4·1% (2·9–5·6)	2·9% (1·7–4·4)
Mild disorder	6·4% (4·4–8·6)	8·5% (6·2–11·1)	6·1% (3·5–9·1)
Disorder without functional impairment	1·4% (0·9–2·0)	6·2% (4·6–7·9)	4·4% (2·7–6·5)
Total	10·8% (8·1–14·2)	21·7% (16·7–28·3)	15·3% (9·9–23·5)

All severity splits taken from Global Burden of Diseases, Injuries, and Risk Factors Study 2016. Disorder without functional impairment indicates cases with disability weight equal to zero once disability attributable to comorbid disorders is portioned out.

**Table 3:** Age-standardised point prevalence with 95% uncertainty intervals, adjusted for comorbidity

	**Depression (without comorbid anxiety disorder)**	**Any anxiety disorder (without comorbid depression)**	Any anxiety disorder with comorbid depression*	**Total**
Severe disorder	0·6% (0·2–1·3)	3·3% (2·1–4·7)	0·4% (0·1–1·0)	4·3% (3·1–5·6)
Moderate disorder	1·1% (0·7–1·5)	2·2% (1·3–3·3)	0·8% (0·5–1·1)	4·0% (2·9–5·5)
Mild disorder	3·7% (2·6–5·1)	6·8% (4·4–9·6)	2·6% (1·9–3·6)	13·0% (10·3–16·2)
Total	5·3% (4·0–6·9)	12·1% (9·4–15·4)	3·8% (2·8–4·9)	21·2% (17·7–24·7)

Estimates of any anxiety disorder include post-traumatic stress disorder. Totals might not equal sum of parts due to rounding.

* Applying a rate of 41·6% (95% uncertainty interval 39·8–43·4) of depression cases with comorbid anxiety. Global Burden of Diseases, Injuries, and Risk Factors Study 2016 severity splits applied.

We only identified two studies that provided epidemiological estimates for psychosis in conflict-affected populations. A cross-sectional study of an internally displaced population in South Darfur reported a prevalence of schizophrenia of 4·1%,^33^ and a general population survey in Timor-Leste reported a schizophrenia point prevalence of 0·34%.^34^ We did not identify any studies that reported epidemiological estimates for bipolar disorder in conflict-affected populations. This small number of studies precluded pooling of estimates, and we conservatively defaulted to global mean prevalence estimates as derived by GDB 2016 for schizophrenia (0·3% [95% UI 0·2–0·3]) and bipolar disorder (0·6% [0·5–0·7]).^35^ Therefore, we were unable to take into account any increase in psychosis or bipolar disorder prevalence in populations affected by conflict.

**Figure 4:**
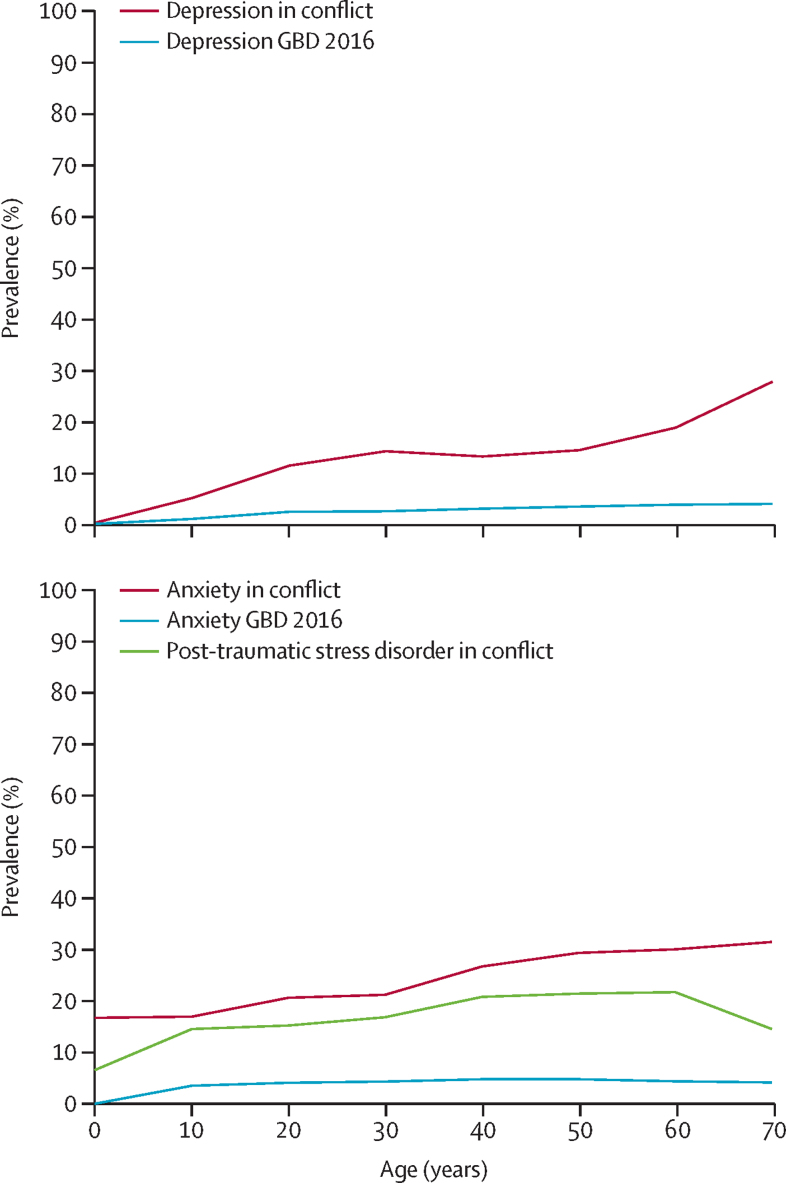
Age-specific prevalence (mean) of depression and any anxiety and post-traumatic stress disorder in conflict-affected populations, 2016 GBD 2016=Global Burden of Diseases, Injuries, and Risk Factors Study 2016.

In conflict settings, trends of depression and anxiety prevalence increased with age. Mean prevalence of post-traumatic stress disorder declined in the older age groups, although there are large ranges of uncertainty surrounding these estimates (figure 4). Our data suggest prevalence of depression, post-traumatic stress disorder, or any anxiety disorder is higher in women than in men, although this finding was only significant for depression (appendix).

Examination of covariate coefficients in our modelling showed that symptom scales significantly overestimate prevalence by about 1·5 to 2 times in conflict-affected populations as compared with diagnostic tools in all three disorder models (appendix).

Heterogeneity in our datasets was large. The median value of the negative binomial model overdispersion parameter calculated by DisMod-MR was 1·2 for anxiety, 0·95 for post-traumatic stress disorder, and 0·96 for depression (where zero is completely uninformative, and infinity is a Poisson distribution).

**Figure 5:**
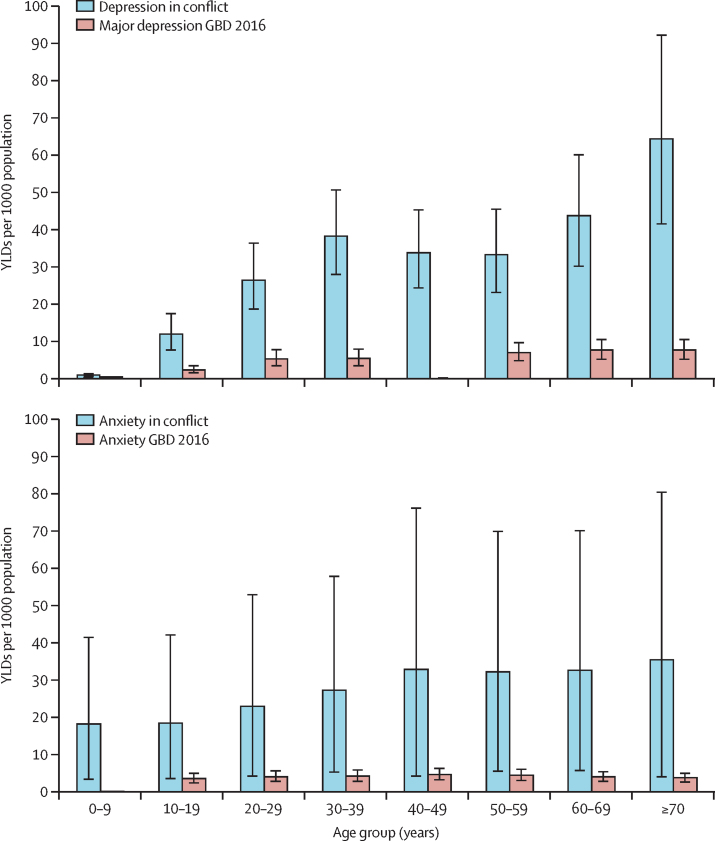
Age-specific years lived with disability (YLDs) per 1000 population (95% uncertainty interval) of depression and any anxiety in conflict-affected populations, 2016 GBD 2016=Global Burden of Diseases, Injuries, and Risk Factors Study 2016.

Age-specific YLDs in conflict-affected populations showed elevated and significant differences across most age groups compared with estimated global YLDs in GBD 2016 (figure 5). We estimated age-standardised YLDs for depression in conflict-affected populations at a rate of 24·8 YLDs per 1000 population (95% UI 16·4–36·0), in contrast to the GBD 2016 global age-standardised estimate of 4·6 YLDs per 1000 population (3·2–6·2). Age-standardised estimates of YLDs for any anxiety disorder in conflict-affected populations were 23·2 YLDs per 1000 population (95% UI 17·0–29·9), as compared with the GBD 2016 estimates of 3·5 YLDs per 1000 population (2·5–4·8).

## Discussion

By updating our previous systematic review on depression and post-traumatic stress disorder^10^ to include more recent data and data on schizophrenia, bipolar, and anxiety disorders, we identified an additional 45 studies published over a 4-year period; this reflects a substantial increase in psychiatric epidemiological research taking place in conflict-affected contexts.

We estimated that approximately one in five people in post-conflict settings has depression, anxiety disorder, post-traumatic stress disorder, bipolar disorder, or schizophrenia. This finding is in contrast to data from GBD 2016,^16^ which suggest a mean global prevalence of one in 14. Our empirically derived estimates show higher prevalence of severe mental disorders than the previous WHO estimates (about 5·1% point prevalence in current estimate compared with 3–4% 12-month prevalence in previous estimates) and higher prevalence of mild to moderate mental disorders (approximately 17% point prevalence in the revised estimates, compared with 15–20% 12-month prevalence in previous estimates). Our estimates of YLDs per 1000 people for depression and post-traumatic stress disorder were more than five times higher than the existing global mean burden of disease estimates. One previous study^10^ reported an age-standardised pooled prevalence of 7·6% for depression and 12·9% for post-traumatic stress disorder.

A useful finding from our study for field researchers who use self-report or symptom-based measures to ascertain mental disorder prevalence estimates is that these instruments were shown to significantly overestimate the prevalence of depression, post-traumatic stress disorder, and anxiety by 1·5 to 2 times. Most of these instruments do not assess clinical significance or function, and hence can overestimate prevalence of disorders compared with diagnostic instruments.

Our study methodology has several strengths. By contrast with previously published reviews, we applied more stringent inclusion and exclusion criteria to our literature search, optimised search strategies, and used updated statistical methods.^9^, ^36^ We sought to address heterogeneity in epidemiological studies by use of Bayesians approaches to allow for a more consistent set of estimates. We made separate estimates for mild, moderate, and severe mental disorders. Although the clinical significance of mild mental disorders in emergencies can be contested,^8^ the clinical needs of people with severe mental disorders are too often neglected.^37^ An important limitation in this study was the raw data. Even with relatively strict inclusion criteria, there was considerable heterogeneity in the mental disorder datasets and their reported estimates, which created large uncertainty around the predicted estimates. This heterogeneity stemmed partly from differences across study designs—an issue inherent to psychiatric epidemiology, particularly research following major emergencies^8^—and partly from the myriad of factors that affect the experience and expression of mental distress in these settings. Many studies failed to report a robust process of translation, cultural adaptation, or validity testing of their instruments. However, a key strength of the DisMod-MR approach is how it addresses heterogeneity through adjustments to the data, which allowed us to create a robust epidemiological profile of mental disorders in conflict-affected populations.

Although not unique to the field of psychiatric epidemiology, issues related to the case definitions of mental disorders warrant consideration in the context of the settings represented in our study. Although reliable systems of classification (DSM and ICD) make it possible to determine prevalence estimates and, therefore, to guide decisions about the development of services, these models of mental disorders assume universality and might not be the most useful way to describe the experience and expression of psychological distress in many of the contexts captured in our study.^38^ Further to the concept of cultural variation are issues presented by shifts in diagnostic criteria. Data included our study are predominantly based on DSM-IV; no studies using DSM-5 were identified. It is apparent that epidemiological research, at least in this context, is yet to move on to most recent version of DSM. In the event of such a transition, it might be prudent to revise anxiety and post-traumatic stress disorder prevalence and YLD estimates.

We only identified two studies on schizophrenia and found no studies on bipolar disorder in conflict-affected populations—too few to pool estimates using meta-regression methods, especially given that one of the studies estimated a ten times higher prevalence estimate than the GBD 2016 prevalence estimate of schizophrenia.^33^ Therefore, we conservatively defaulted to global mean prevalence estimates as derived by GDB 2016.^16^ The estimates for psychosis we report here might thus be underestimates and do not take into account the studies we had to exclude from our systematic search that suggest an increase in psychoses in populations affected by conflict.^39^ Because of the paucity of data, we had to use several assumptions and proxy inputs—such as a comorbidity adjustment informed by a single study from a conflict-affected population and the proxy use of GBD 2016 disability weights—which should be considered when interpreting our findings, until more and better-quality epidemiological data become available. Furthermore, the study did not include comorbid disorders, such as alcohol use disorders and epilepsy, which are frequently addressed within mental health programmes.^14^

Nonetheless, our study identified the sustained presence of high prevalence of mental disorders in conflict-affected countries, making a compelling case for global humanitarian, development, health, and mental health communities to prioritise development of mental health services in conflict and post-conflict settings.

Evidence for building systems for mental health care after conflict shows that emergencies, which can generate political interest and funding for mental health, can be a catalyst for the meaningful development of mental health care.^3^ A review of lessons learned from such work in ten countries showed that focusing on system-wide reform to address both new-onset and pre-existing mental disorders is crucial.^3^ Practical guidance for management of disorders that should be scaled up in conflict-affected countries already exists. WHO and UN High Commissioner for Refugees have designed the mhGAP Humanitarian Intervention Guide,^14^ which addresses the assessment and management of moderate and severe mental disorders in non-specialised health-care settings, such as general hospitals and primary health care. Moreover, a variety of packages designed to address multiple mental disorders, such as Problem Management Plus, Common Elements Treatment Approach and Self-Help Plus, have been used with promising results among conflict-affected Pakistanis, Burmese refugees, and South Sudanese refugees.^40^, ^41^, ^42^ It should be noted that there is wide consensus that mental health and psychosocial support for affected populations should go beyond psychological and medical treatments for mental disorders, and that such support should include psychosocial intervention that strengthens community self-help and support^13^ and advocacy for security and protection and for adequate humanitarian aid, including basic health services and livelihood support.

Our findings highlight the need to prioritise conflict-affected countries for implementation of the WHO Mental Health Action Plan.^2^ This will require a focus on investment in leadership and governance for mental health, and the development of integrated, responsive mental health and social care services in community-based settings. Strategies for promotion and prevention in mental health, and building and strengthening of information systems, evidence, and research for mental health in conflict-affected countries, are also needed. These services could be initiated with short-term emergency funds that are often available during crises. Demonstration projects can provide proof of concept and attract the further support and funds necessary for system development to reduce the burden of mental disorders among people affected by war and other conflict.^3^

Our study shows that the impact of conflict on people's mental health is higher than previous estimates suggest. Mental health care must be prioritised in countries affected by conflict, not least for the well established links between mental health, functioning, and country development.
