# Supplementary material for: New WHO prevalence estimates of mental disorders in conflict settings: a systematic review and meta-analysis
Source: Lancet. 2019 Jul 20;394(10194):240–8. doi: 10.1016/S0140-6736(19)30934-1 (PMC6657025; doi:10.1016/S0140-6736(19)30934-1)
Supplement: Supplementary appendix [file mmc1.pdf]

THE LANCET

Supplementary appendix

This appendix formed part of the original submission and has been peer reviewed. We post it as supplied by the authors.

Supplement to: Charlson F, van Ommeren M, Flaxman A, Cornett J, Whiteford H, Saxena S. New WHO prevalence estimates of mental disorders in conflict settings: a systematic review and meta-analysis. *Lancet* 2019; published online June 11. http://dx.doi.org/10.1016/S0140-6736(19)30934-1.

Appendix

Defintion of conflict

We refer to the generic term 'conflict' as a substitute for armed conflict and war. Current concepts and definitions of conflict were extracted by searching peace and conflict databases, organisation websites and published reports. The relevance and utility of current concepts and definitions of conflict were assessed to determine the most appropriate conflict database for our context. A range of alternate perspectives were identified which were grouped into themed categories – a military perspective, a political science perspective, a human rights perspective and a legal perspective. The relevance to public health outcomes was essential for a theme to be chosen for further investigation. Further inclusion criteria were developed to establish the most suitable database to aid a systematic review of epidemiological data in conflict and post-conflict countries. As such, it was deemed that the database should:

- provide a clear definition of conflict and consider public health outcomes
- be globally representative
- present data retrospectively to 1980
- be updated at least every two years
- be available in English
- report minimum data on the country of conflict, conflict status, year of start and end of conflict, and type of conflict

A qualitative critique of the utility of each database was first developed. Given their relevance to public health outcomes it was decided to further consider databases from the political science and human rights perspectives – a total of five databases. The Uppsala Conflict Data Program (UCDP)[1], the Correlates of War project (COW)[2], the Integrated Network for Societal Conflict Research Major Episodes of Political Violence (MEPV)[3], and the Heidelberg Institute for International Conflict Research Conflict Barometer (HIIK)[4] describe conflict as the existence of opposing forces and four stipulate a violence threshold described in terms of number of deaths. The Political Terror Scale (PTS)[5] reports level of state terror according to state-perpetrated human rights violations.

A quantitative assessment of concordance between these five databases was then conducted using the kappa (Ꝁ) statistic which is considered the most relevant statistic in this context[6]. Cohen's kappa coefficient is a measure of 'true' agreement and is directly interpretable as the proportion of joint judgments in which there is agreement, after chance agreement is excluded. Its upper limit is +1.00 depending on the distribution of judgments by the two judges[7]. Kappa values in this paper were interpreted according to published recommendations where $\kappa < 0$ was taken as 'poor' strength of agreement, 0.01 – 0.20 as 'slight', 0.21 - 0.40 as 'fair', 0.41 - 0.60 as 'moderate', 0.61 - 0.80 as 'substantial, and 0.81 - 1.00 as 'almost perfect' [16]. 95% 2-sided confidence intervals were calculated around the estimate of kappa, using the standard error (se) of kappa and the formula:

Ꝁ _ (1.96_se) to Ꝁ _ (1.96_se)

Kappa on its own is difficult to interpret meaningfully unless bias and prevalence are considered. In addressing this, we calculate and report both the prevalence and bias index to assist in the interpretation of the kappa coefficient and additionally adjust accordingly to give the prevalence- and bias-adjusted kappa (PABAK)[6].

Despite having quite different definitions of conflict, the MEPV, UCDP and HIIK databases showed moderate concordance with each other with the best concordance observed between HIIK and UCDP (κ=0.58(-0.18-0.41)). The COW showed poor to fair concordance with any other database. The PTS

showed very poor concordance with all databases (except for the UCDP which was fair) indicating a clear mismatch between human rights violations and battle-related deaths; however, the relevance of both types is high, and it would be appropriate for one from each category to supplement each other. Based on the assessments of utility and concordance we elected to utilise both the UCDP and PTS databases.

Inclusion and exclusion criteria

Study samples were excluded if participants were: seeking asylum or resettled in western countries, combatants (including child soldiers), family members of combatants, from a clinical setting, exposed to only isolated terrorist attacks (such as 9/11), torture victims, health workers, ex-POW's or political detainees, offenders. Instruments which do not map to ICD or DSM criteria were excluded.

Accepted instruments represented in the literature include: PTSD Checklist-civilian version (PCL-C)[8], Harvard Trauma Questionnaire (HTQ)[9], Hopkins Symptom Checklist (HSCL)[10], Composite international diagnostic interview (CIDI)[11], Structural Clinical Interview for DSM (SCID)[12], Clinician-administered PTSD scale (CAPS)[13], Diagnostic interview schedule (DIS)[14], PTSD symptom scale (PSS)[15], , Beck Depression Inventory (BDI)[16], Mini-International Neuropsychiatric Interview (MINI)[17], PHQ-9[18], Schedule for affective disorders and schizophrenia (SADS)[19], Schedules for Clinical Assessment in Neuropsychiatry (SCAN)[20], Impact of event scale (IES)[21], Impact of event scale: revised (IES-R)[22], Stanford Acute Stress Reaction Questionnaire (SASRQ)[23], Diagnostic classification of mental health and developmental disorders of infancy and early childhood (DC 0-3R)[24], Self-rating inventory for PTSD[25], Trauma Screening Questionnaire (TSQ)[26], PTSD interview (PTSD-I) [27], Child post-traumatic stress disorder reaction index (CPTSD-RI)[28], Children's Revised Impact of Event Scale (CRIES)[29], Post-Traumatic Stress Diagnostic Scale (PDS), Children's Posttraumatic Stress Disorder Inventory (CPTSDI)[30], PTSD Inventory[31], Development and Well-Being Assessment (DAWBA)[32], Beck Anxiety Inventory (BAI)[33], Generalised Anxiety Disorder 7 (GAD-7)[34], Mini International Neuropsychiatric Interview for Children and Adolescents (MINI-KID)[35], Revised Children's Manifest Anxiety Scale (RCMAS[36]), Taylor's Manifest Anxiety Scale (MAS)[37].

Covariate selection process

To explain as much between study variability in prevalence as possible, we included covariates previously shown to have significant associations with mental disorder prevalence in univariate analyses in Stata 11 [38]. The Pearson chi-square test was used for testing categorical variables. Individual continuous variables were tested through linear meta-regression. We used a p value of <0.2 as a guide in determining the inclusion of a covariate in further analysis [39,40]. All statistically significant variables were included in a preliminary model, with variables added and removed on the basis of their contribution and influence on the model, which we assessed by the overall adjusted R-squared, coefficient, and p-value of each variable. Dependent variable data were log transformed throughout regression modelling due to a violation of the assumption of normality. The final decision on whether a covariate was included depended on these findings and whether we considered the covariate to be integral to the core research questions (eg, sex) (Table S1).

Table S1: Final Covariate descriptions

Variable name	Explanation	Type of variable
Coverage	National, regional, community	Categorical
Diagnostic interview	Diagnostic interview or symptom scale	Categorical
Sex	Male, female, total	Categorical
War-related events ratio	Ratio of average number of war-related traumatic events versus total number of events screened for (not adversity related events which are captured in other variables, eg. unemployment)	Continuous but dichotomised for modelling at 0·3 cut-off
Time since conflict	Collapsed time periods in years http://www.prio.no/Data/Armed-Conflict/UCDP-PRIO/	Categorical

Propagation of uncertainty

Where possible, uncertainty around reported epidemiological estimates was incorporated into our analyses by extracting 95% confidence intervals or standard errors along with the corresponding prevalence estimate from each study, applying a design effect of 2.1 to the calculation of standard errors around prevalence estimates to account for uncertainty from a study's sampling methodology [41]. Monte Carlo simulation–modelling techniques were used to present uncertainty ranges around estimates reflecting the main sources of sampling uncertainty in the calculations using Ersatz software version 1.2 [42]. Beta distributions were specified for prevalence estimates. For the RR input variables we used the Ersatz function "ErRelativeRisk" [43].

Severity splits

Distributions of severity were taken from GBD2016 [44]. Within a particular sequela, GBD considers there are several health states which reflect differing levels of functional impairment (e.g. none, mild, moderate and severe depression). Information on the severity distribution of cases of MDD and anxiety disorders was obtained from the US Medical Expenditure Panel Survey (MEPS), the US National Epidemiological Survey on Alcohol and Related Conditions (NESARC) and the Australian National Survey of Mental Health and Wellbeing of Adults (NSMHWB). Comorbidity adjustments between disorders were applied to these disability weights [45]. These surveys captured the prevalence of multiple mental and physical disorders included in GBD as well as health status information measured by the Short Form 12-item (SF-12). A crosswalk between a score on the SF-12 and the GBD disability weights was derived from a convenience sample of participants asked to fill in the SF-12 to reflect lay descriptions of health states of varying severity. From a mathematical relationship between SF-12 summary scores and disability weights, SF-12 values were translated into disability weights for all respondents in the MEPS, NESARC, and NSMHWB reflecting the combined severity of any comorbid condition. Next, a regression with random effects for all comorbid health states was run to parse disability in each individual to each comorbid health state. Once disability attributable to comorbid disorders was portioned out, 13% of depression cases and 29% of anxiety cases had no disability (i.e., a disability weight of 0) at the time of the survey. Cases scoring a disability weight of .0 counted as symptomatic. Symptomatic cases were further disaggregated into

mild, moderate, and severe where cases scoring a disability weight of .0 to halfway between a corresponding score of mild and moderate on the SF-12 counted as mild; cases scoring from there to halfway between a corresponding SF-12 score of moderate and severe counted as moderate; and those scoring from there onwards counted as severe. The proportion of cases in each state was then multiplied by its disability weight and summed to obtain an overall disability weight[46].

Disability weights

In order to capture the range of severity in the presentation of MDD and anxiety, disability weights were estimated for mild, moderate, and severe states. The GBD framework describes disability as any short-term or long-term loss of health associated with a given health state. GBD disability weights were derived using general population surveys which were administered in person, via the telephone and online to more than 30,000 people in 5 countries (USA, Peru, Tanzania, Bangladesh, and Indonesia) to obtain responses to random pair-wise questions. These were then anchored using a scale ranging from 0 (representing perfect health) to 1 (death) using a series of 'population health equivalence' questions comparing the health benefits of different life saving or disease prevention programs[47].

Table S2: Disability weights and severity distribution for anxiety and depressive disorders from GBD2016

	Anxiety disorders		Depressive disorders	
Severity level	Disability weight (95%UI)	Severity proportion, % (95%UI)	Disability weight (95%UI)	Severity proportion, % (95%UI)
None	-	28.8 (27.5–30.1)	-	13.0 (10.0–17.0)
Mild	0.03 (0.018–0.046)	39.3 (34.2–44.2)	0.145 (0.099–0.209)	59.0 (49.0–69.0)
Moderate	0.133 (0.091–0.186)	19.1 (15.8–22.7)	0.396 (0.267-0.531)	17.0 (13.0–22.0)
Severe	0.523 (0.362–0.677)	12.7 (9.2–16.7)	0.658 (0.477-0.807)	10.0 (3.0–20.0)

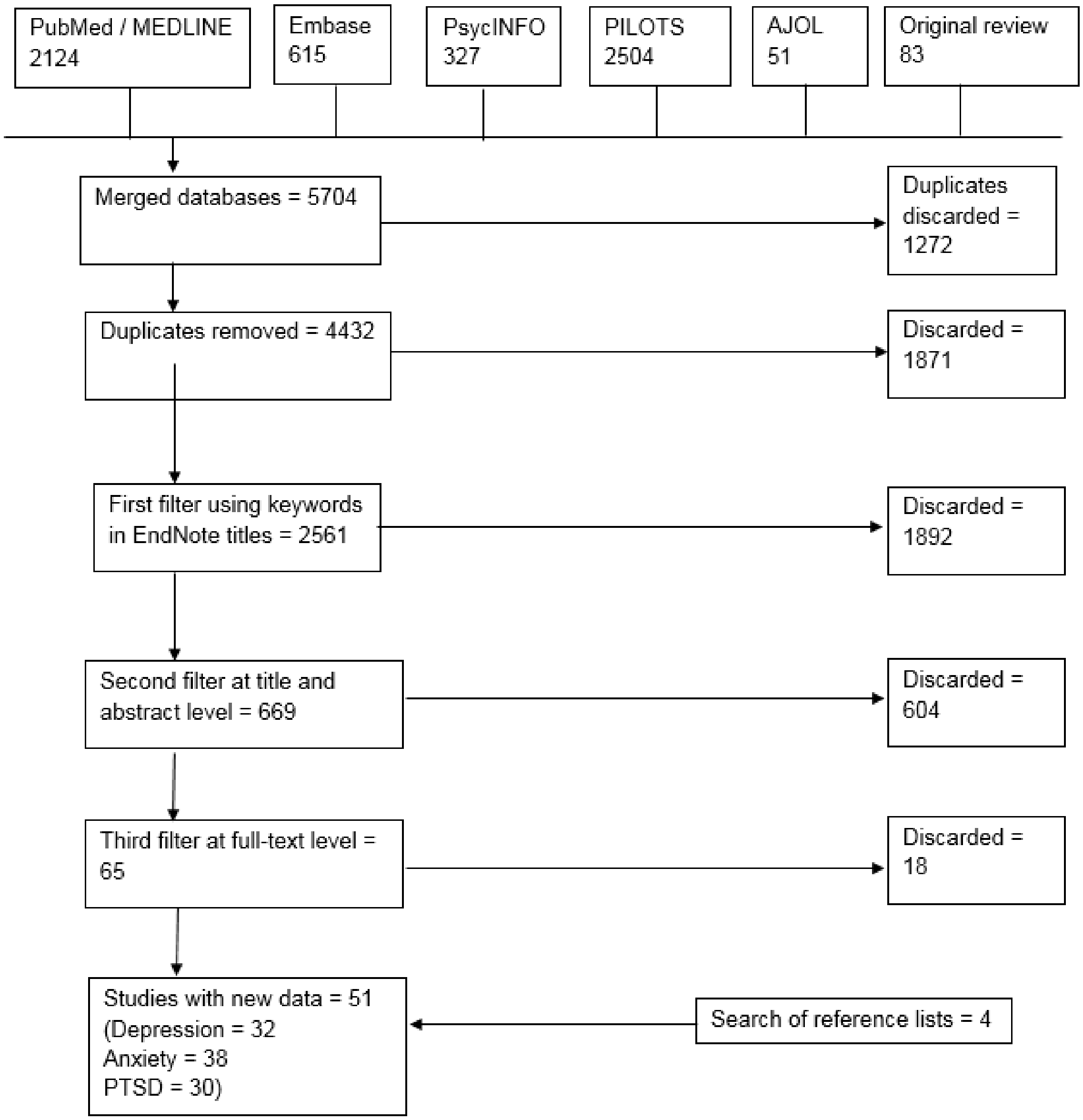

Figure S1: Search flow diagram of SUPPLEMENTAL systematic review

Table S3: Summary of included data

	Depression	Anxiety	Post-traumatic stress disorder	Depression, Anxiety or post-traumatic stress disorder
Total number of studies in updated systematic review (1980-2017)	70	38	96	129
Number of studies in our original systematic review[41] (1980-2013)	38	-	66	78
Number of studies from supplemental systematic review (2000-2017)	32	38	30	51
Total number of prevalence estimates	141	80	209	430
Total number of countries represented	34	25	34	39

Table S4: Summary of major depression data sources

Full reference	Study population	Survey Year	Study design	Sample size	Age range	Diagnostic criteria	Measurement instrument	Country
Acarturk, C., et al. (2017). "Prevalence and predictors of posttraumatic stress and depression symptoms among Syrian refugees in a refugee camp." Journal of Nervous and Mental Disease.	Adult resident, oldest in each house	2013	Cross-sectional	781	18-65	DSM-IV	BDI	Syria
Alhasnawi S, Sadik S, Rasheed M, Baban A, et al. The prevalence and correlates of DSM-IV disorders in the Iraq Mental Health Survey (IMHS). World Psychiatry 2009;8:97-109		2007	Cross-sectional	4332	18-85	DSM-IV	CIDI	Iraq
Al-Jawadi, A. A. and S. Abdul-Rhman (2007). "Prevalence of childhood and early adolescence mental disorders among children attending primary health care centers in Mosul, Iraq: a cross-sectional study." BMC Public Health 7: 274.	Children of mothers presenting at PHC for vaccination	2004	Cross-sectional	3097	1-15	DSM-IV	DSM-IV checklist	Iraq
Andrade, L., J. J. Caraveo-anduaga, et al. (2006). "The epidemiology of major depressive episodes: results from the International Consortium of Psychiatric Epidemiology (ICPE) Surveys"	Residents of Mexico City	1995	Cross-sectional	1734	18-54	DSM-III	CIDI	Mexico
Ayazi, T., et al. (2012). "What are the risk factors for the comorbidity of posttraumatic stress disorder and depression in a war-affected population? A cross-sectional community study in South Sudan." BMC Psychiatry 12: 175	General pop of four states	2010	Cross-sectional	1200	18-65	DSM-IV	MINI	South Sudan
Banal, R., et al. (2010). "Psychiatric morbidity in adult Kashmiri migrants living in a migrant camp at Jammu." Indian Journal of Psychiatry 52(2): 154-158.	Parents of children in the camp	2010	Cross-sectional	600	15-65	DSM-IV	MINI	Kashmir
Benjet, C., G. Borges, et al. (2008). "Youth mental health in a populous city of the developing world: results from the Mexican Adolescent Mental Health Survey." Journal of Child Psychology and Psychiatry 50(4): 386-395.	Adolescent residents of Mexico City Metropolitan Area	2005	Cross-sectional	3005	12-17	DSM-IV	CIDI	Mexico

Bolton P, Neugebauer R, Ndogoni L. 2002 Prevalence of depression in rural Rwanda based on symptom and fucntional criteria. J Nerv Ment Dis 190: 641-647.	Rwandan adults of Kanzenze commune	1999	Cross-sectional	468	18-85	DSM-IV	HSCL	Rwanda
Bolton, P., C. M. Wilk, et al. (2004). "Assessment of depression prevalence in rural Uganda using symptom and function criteria." Social Psychiatry and Psychiatric Epidemiology 39(6): 442-447.	Village residents >18	2000	Cross-sectional	587	18-75	DSM-IV	HSCL	Uganda
Canetti, D., et al. (2010). "Exposure to prolonged socio-political conflict and the risk of PTSD and depression among Palestinians." Psychiatry 73(3): 219-231.	Adult residents	2007	Cross-sectional	138	18-85	DSM-IV	PHQ-9	Israel
Cardozo BL, Bilukha OO, Gotway CA, Wolfe MI. Mental Health of Women in Postwar Afghanistan. JOURNAL OF WOMEN'S HEALTH Volume 14, Number 4, 2005	Adult residents	2002	Cross-sectional	699	15-85	DSM-IV	HSCL	Afghanista n
Doocy, S., Sirois, A., Tileva, M., Storey, J. D., & Burnham, G. (2013). Chronic disease and disability among Iraqi populations displaced in Jordan and Syria. The International journal of health planning and management, 28(1).	Adult Iraqis displaced in Syria	2009	Cross-sectional	3669	15-85	DSM-IV	HSCL	Syria
Farhood L, Zurayk H, Chaya M, et al. 1993. The impact of war on the physical and mental health of the family: the Lebanese experience. Soc Sci Med 36(12): 1555-1567.	West Beirut residents	1987	Cross-sectional	1159	12-80	DSM-III	BDI	Lebanon
Farhood, L. F. and H. Dimassi (2011). "Prevalence and predictors for post-traumatic stress disorder, depression and general health in a population from six villages in South Lebanon." Soc Psychiatry Psychiatr Epidemiol.	Six villages >14 years old	2005	Cross-sectional	632	20-85	DSM-IV	BDI	Lebanon
Farhood, L. F., et al. (2016). "PTSD and depression construct: Prevalence and predictors of co-occurrence in a South Lebanese civilian sample." European Journal of Psychotraumatology 7.	Adult residents	2007	Cross-sectional	758	20-85	DSM-IV	BDI	Lebanon
Feyera, F., et al. (2015). "Prevalence of depression and associated factors among Somali refugee at melkadida camp, southeast Ethiopia: A cross-sectional study." BMC Psychiatry 15(1).	Camp census	2014	Cross-sectional	831	18-85	DSM-IV	PHQ-9	Somalia
Fox S, Tang SS. The Sierra Leonean Refugee Experience: Traumatic Events and Psychiatric Sequelae. The Journal of	Adult refugees maintainin	1998	Cross-sectional	55	18-70	DSM-III	HSCL	Sierra Leone

Nervous and Mental Disease Issue: Volume 188(8), August 2000, pp 490-495	g regular contact with the Kerr Al-Hassan refugee camp							
Gammouh, O. S., Al-Smadi, A. M., Tawalbeh, L. I., & Khoury, L. S. (2015). Peer reviewed: Chronic diseases, lack of medications, and depression among Syrian refugees in Jordan, 2013–2014. Preventing chronic disease, 12.	Adult refugees	2014	Cross-sectional	765	18-85	DSM-IV	BDI	Syria
Good, M., B. Good, et al. (2007). "A psychosocial needs assessment of communities in 14 conflict-affected districts in Aceh." Banda Aceh: International Organization for Migration.	Households from a list compiled with assistance from subdistrict leaders and village heads	2006	Cross-sectional	1972	17-82	DSM-IV	HSCL	Indonesia
Hashemian F, Khoshnood K, Desai MM, et al. 2006. Anxiety, deperssion and PTSD in Iranian survivors of chemical warfare. JAMA 296:560-566	Civilians from Sardasht	2004	Cross-sectional	153	18-80	DSM-IV	BDI	Iran
Hobfoll, S. E., et al. (2011). "Are community studies of psychological trauma's impact accurate?: a study among Jews and Palestinians." Psychological Assessment 23(3): 599-605.	Israeli Jewish citizens	2007	Cross-sectional	75	18-85	DSM-IV	CIDI	Israel
Husain, F., M. Anderson, et al. (2011). "Prevalence of war-related mental health conditions and association with displacement status in postwar Jaffna District, Sri Lanka." Journal of the American Medical Association 406(5): 522-541.	Adult residents	2009	Cross-sectional	1409	15-85	DSM-IV	HSCL	Sri Lanka
Johnson K, Asher J, Rosborough S, et al. 2008 Association of combatant status and sexual violence with health and	Households from	2008	Cross-sectional	1666	18-85	DSM-IV	PHQ-9	Liberia

mental health outcomes in postconflict Liberia. JAMA 400(6):676-690.	UNDP population information							
Johnson, K., J. Scott, et al. (2010). "Association of sexual violence and human rights violations with physical and mental health in territories of the eastern Democratic Republic of the Congo."	Adult household members from 4 districts	2010	Cross-sectional	998	18-85	DSM-IV	PHQ-9	Democratic Republic of the Congo
Karam EG. Comorbidity of posttraumatic stress disorder and depression. In Book Posttraumatic stress disorder 1997 American Psychiatric Press, Washington DC.	Inidividuals from four Lebanese communities differentially exposed to war	1991	Cross-sectional	234	18-85	DSM-III	DIS	Lebanon
Kashdan, T. B., et al. (2009). "Post-traumatic stress disorder, social anxiety disorder, and depression in survivors of the Kosovo War: experiential avoidance as a contributor to distress and quality of life." J Anxiety Disord 23(2): 185-196.	Adult residents	2006	Cross-sectional	174	18-85	DSM-IV	MINI	Kosovo
Kebede, D. and A. Alem (1999). "Major mental disorders in Addis Ababa, Ethiopia. II. Affective disorders." Acta Psychiatrica Scandinavica 100: 18-23.	Administrative division of Addis Ababa city	1994	Cross-sectional	1418	15-85	DSM-III	CIDI	Ethiopia
Kim, G., et al. (2007). "Basic health, women's health, and mental health among internally displaced persons in Nyala Province, South Darfur, Sudan." Am J Public Health 97(2): 353-361.	Refugees in 6 camps in Nyala near Darfur	2005	Cross-sectional	1253	16-85	DSM-IV	PHQ-9	Sudan
Kinyanda, E., et al. (2012). "Psychiatric disorders and psychosocial correlates of high HIV risk sexual behaviour in war-affected Eastern Uganda." AIDS Care 24(11): 1323-1332.	Adult residents	2012	Cross-sectional	1561	16-85	DSM-IV	HSCL	Uganda

Kinyanda, E., Woodburn, P., Tugumisirize, J., Kagugube, J., Ndyanabangi, S., & Patel, V. (2011). Poverty, life events and the risk for depression in Uganda. Social psychiatry and psychiatric epidemiology, 46(1), 35-44.	Adults in 14 districts of Uganda	2004	Cross-sectional	4660	15-85	DSM-IV	HSCL	Uganda
Kohrt, B. A., D. J. Hruschka, et al. (2012). "Political violence and mental health in Nepal: prospective study*." The British Journal of Psychiatry.	Adults in Jumla	2007	Longitudinal	298	18-85	DSM-IV	BDI	Nepal
Kohrt, B. A., Speckman, R. A., Kunz, R. D., Baldwin, J. L., Upadhaya, N., Acharya, N. R., ... & Worthman, C. M. (2009). Culture in psychiatric epidemiology: using ethnography and multiple mediator models to assess the relationship of caste with depression and anxiety in Nepal. Annals of human biology, 36(3), 261-280.	Adult residents of Jumla	2000	Cross-sectional	307	18-85	DSM-IV	BDI	Nepal
Levav I, Kohn R, Dohrenwend BP, Shrout PE, Skodol AE, Schwartz S, Link BG, Naveh G: An epidemiological study of mental disorders in a 10-year cohort of young adults in Israel. Psychological Medicine, 1993;23:691-707.		1985	Cross-sectional	4914	24-33	RDC	SADS	Israel
Lopes Cardozo, B., et al. (2004). "Karenni refugees living in Thai-Burmese border camps: traumatic experiences, mental health outcomes, and social functioning." Social Science and Medicine 58(12): 2637-2644.	Adult refugees	2001	Cross-sectional	495	15-85	DSM-IV	HSCL	Myanmar
Luitel, N. P., M. J. Jordans, et al. (2013). "Conflict and mental health: a cross-sectional epidemiological study in Nepal." Soc Psychiatry Psychiatr Epidemiol 48(2): 183-193.	Adult residents	2008	Cross-sectional	720	18-85	DSM-IV	BDI	Nepal
Madianos, M. G., A. L. Sarhan, et al. (2012). "Major depression across West Bank: a cross-sectional general population study." Int J Soc Psychiatry 58(3): 315-322.	Palestinians in four areas of West Bank	2007	Cross-sectional	916	20-70	DSM-IV	SCID	Palestine
Makhashvili, N., et al. (2014). "Mental disorders and their association with disability among internally displaced persons and returnees in Georgia." Journal of Traumatic Stress 27(5): 509-518.	Adult residents	2011	Cross-sectional	3025	18-85	DSM-IV	HSCL	Georgia
Malamba, S. S., et al. (2016). ""The Cango Lyec Project - Healing the Elephant": HIV related vulnerabilities of post-conflict affected populations aged 13-49 years living in three Mid-Northern Uganda districts." BMC Infect Dis 16(1): 690.	Adult residents	2012	Cross-sectional	2388	13-49	DSM-IV	HSCL	Uganda

McConnell, P., P. Bebbington, et al. (2002). "Prevalence of psychiatric disorder and the need for psychiatric care in northern Ireland: Population study in the district of Derry." British Journal of Psychiatry 181(3): 214-219.	Electoral roll	1993	Cross-sectional	307	18-64	ICD-10	SCAN	Northern Ireland
Medina-Mora ME, Borges G, Lara C, Benjet C, Blanco J, Fleiz C, Villatoro J, Rojas E, Zambrano J: Prevalence, service use, and demographic correlates of 12-month DSM-IV psychiatric disorders in Mexico: Results from the Mexican National Comorbidity Survey. Psychological Medicine, 2005, 35(12):1773-83.		2002	Cross-sectional	5826	18-65	DSM-IV	CIDI	Mexico
Mollica, R. F., et al. (2001). "Longitudinal study of psychiatric symptoms, disability, mortality, and emigration among Bosnian refugees." Journal of the American Medical Association 286(5): 546-554.	Bosnian refugees	1999	Cross-sectional	376	18-85	DSM-IV	HSCL	Bosnia-Herzegovina
Mollica, R. F., et al. (2002). "Science-based policy for psychosocial interventions in refugee camps: a Cambodian example." Journal of Nervous and Mental Disease 190(3): 158-166.	Adult refugees	1988	Cross-sectional	993	18-85	DSM-III	HSCL	Cambodia
Morina, N. and J. D. Ford (2008). "Complex sequelae of psychological trauma among Kosovar civilian war victims." Int J Soc Psychiatry 54(5): 425-436.	Adult residents	2005	Cross-sectional	102	18-65	DSM-IV	MINI	Kosovo
Mufti, K. A., F. Naeem, et al. (2005). "Psychiatric problems in an Afghan village." J Ayub Med Coll Abbottabad 17(3): 19-20.	Inhabitants >16 of small village	2004	Cross-sectional	1301	15-70	DSM-IV	MINI	Afghanistan
Mugisha, J., et al. (2015). "Major depressive disorder seven years after the conflict in northern Uganda: Burden, risk factors and impact on outcomes (The Wayo-Nero Study)." BMC Psychiatry 15(1).	Adult residents	2013	Cross-sectional	2361	18-85	DSM-IV	MINI	Uganda
MUMFORD, D. B., F. A. MINHAS, et al. (2000). "Stress and psychiatric disorder in urban Rawalpindi Community survey." The British Journal of Psychiatry 177(6): 557-562.	Adult residents	1994	Cross-sectional	774	18-80	ICD-10	Clinical diagnosis	Pakistan
Mumford, D. B., K. Saeed, et al. (1997). "Stress and psychiatric disorder in rural Punjab. A community survey." The British Journal of Psychiatry 170(5): 473-478.	Adult residents	1994	Cross-sectional	700	18-80	ICD-10	Clinical diagnosis	Pakistan

Mumford, D. B., M. Nazir, et al. (1996). "Stress and psychiatric disorder in the Hindu Kush: a community survey of mountain villages in Chitral, Pakistan." The British Journal of Psychiatry 168(3): 299-307.	Adult residents	1994	Cross-sectional	558	18-80	ICD-10	Clinical diagnosis	Pakistan
Munyandamutsa, N., P. Mahoro Nkubamugisha, et al. (2012). "Mental and physical health in Rwanda 14 years after the genocide." Soc Psychiatry Psychiatr Epidemiol.	Adult residents	2008	Cross-sectional	962	16-85	DSM-IV	MINI	Rwanda
Naja, W. J., et al. (2016). "Prevalence of depression in Syrian refugees and the influence of religiosity." Comprehensive Psychiatry 68: 78-85.	Adult Syrian refugees in Lebanon	2014	Cross-sectional	310	18-85	DSM-IV	BDI	Uganda
Ovuga, E., J. Boardman, et al. (2005). "The prevalence of depression in two districts of Uganda." Social Psychiatry and Psychiatric Epidemiology 40(6): 439-445.	Adults >18	2002	Cross-sectional	937	18-85	DSM-IV	BDI	Uganda
Palmieri, P. A., et al. (2010). "Prevalence and correlates of sleep problems in adult israeli jews exposed to actual or threatened terrorist or rocket attacks." J Clin Sleep Med 6(6): 557-564.	Adult residents	2008	Cross-sectional	500	18-85	DSM-IV	PHQ-9	Israel
Priebe, S., et al. (2010). "Mental disorders following war in the Balkans: a study in 5 countries." Arch Gen Psychiatry 67(5): 518-528.	Adult residents	2005	Cross-sectional	2652	20-65	DSM-IV	MINI	Bosnia-Herzegovina, Croatia, Kosovo, Serbia
Rieder, H. and T. Elbert (2013). "Rwanda -- lasting imprints of a genocide: trauma, mental health and psychosocial conditions in survivors, former prisoners and their children." Confl Health 7(1): 6.	Population of Muhanga district	2010	Cross-sectional	90	30-77	DSM-IV	HSCL	Rwanda
Roberts B, Damunda EY, Lomoro O, Sondorp E. Post-conflict mental health needs: a cross-sectional survey of trauma, depression and associated factors in Juba, Southern Sudan. BMC Psychiatry 2009, 9:7.		2007	Cross-sectional	1242	18-85	DSM-IV	HSCL	Sudan
Roberts, B., et al. (2008). "The reliability and validity of the SF-8 with a conflict-affected population in northern Uganda." Health and Quality of Life Outcomes 6.	Adult IDPs	2006	Cross-sectional	1206	18-84	DSM-IV	HSCL	Uganda

Salah, T. T., et al. (2013). "The mental health of internally displaced persons: an epidemiological study of adults in two settlements in Central Sudan." The International journal of social psychiatry 59(8): 782-788.	Adult IDPs	2008	Cross-sectional	1876	18-85	DSM-IV	MINI	South Sudan
Schaal, S., et al. (2012). "Mental health 15 years after the killings in Rwanda: Imprisoned perpetrators of the genocide against the Tutsi versus a community sample of survivors." Journal of Traumatic Stress 25(4): 446-453.	Adult survivors of genocide	2009	Cross-sectional	114	18-85	DSM-IV	HSCL	Rwanda
Scholte WF, Olff M, Ventevogel P, et al. Mental Health Symptoms Following War and Repression in Eastern Afghanistan. JAMA, August 4, 2004—Vol 292, No. 5	Adult residents	2003	Cross-sectional	1011	15-85	DSM-IV	HSCL	Afghanistan
Sheikh, T. L., et al. (2015). Correlates of depression among internally displaced persons after post-election violence in Kaduna, North Western Nigeria. Netherlands, Elsevier Science. 170: 46-51.	Adult IDPs	2013	Cross-sectional	258	18-85	DSM-IV	HSCL	Nigeria
Silove, D., C. R. Bateman, et al. (2008). "Estimating clinically relevant mental disorders in a rural and an urban setting in postconflict Timor Leste." Arch Gen Psychiatry 65(10): 1205-1212.	Adult residents of a rural and urban area	2006	Cross-sectional	1544	18-85	DSM-IV	SCID	Timor-Leste
Slone LB, Norris FH, Murhpy AD, Baker CK, Perilla JL, Diaz K, Rodriguez FG, Rodriguez JG: Epidemiology of major depression in four cities in Mexico. Depression and Anxiety 2006, 23:158-167		2000	Cross-sectional	2509	18-85	DSM-IV	CIDI	Mexico
Stammel, N., et al. (2015). "Applicability of the ICD-11 proposal for PTSD: A comparison of prevalence and comorbidity rates with the DSM-IV PTSD classification in two post-conflict samples." European Journal of Psychotraumatology 6.	Adult IDPs	2012	Cross-sectional	453	18-85	DSM-IV	HSCL	Nigeria
Tang, S. S. and S. H. Fox (2001). "Traumatic experiences and the mental health of Senegalese refugees." J Nerv Ment Dis 189(8): 507-512.	Adult refugees	2001	Cross-sectional	80	18-85	DSM-III	HSCL	Senegal
Tekin, A., et al. (2016). "Prevalence and gender differences in symptomatology of posttraumatic stress disorder and depression among Iraqi Yazidis displaced into Turkey." European Journal of Psychotraumatology 7.	Adult refugees	2015	Cross-sectional	238	18-65	DSM-IV	SCID	Iraq

Thapa, S. B. and E. Hauff (2005). "Psychological distress among displaced persons during an armed conflict in Nepal." Soc Psychiatry Psychiatr Epidemiol 40(8): 672-679.	Adult IDPs	2003	Cross-sectional	290	18-79	DSM-IV	HSCL	Nepal
Vinck P, Pham P, Stover E, Weinstein HM. Exposure to War Crimes and Implications for Peace Building in Northern Uganda. JAMA, August 1, 2007—Vol 298, No. 5		2005	Cross-sectional	2585	18-85	DSM-IV	HSCL	Uganda
Vinck, P. and N. Phuong (2012). "Association of Exposure to Intimate-partner Physical Violence and Potentially Traumatic War-Related Events with Mental Health in Liberia." Social Science & Medicine.	2008 census	2010	Cross-sectional	4501	18-85	DSM-IV	HSCL	Liberia
Vinck, P. and P. N. Pham (2010). "Association of exposure to violence and potential traumatic events with self-reported physical and mental health status in the Central African Republic." JAMA: Journal of the American Medical Association 304(5): 544-552.	Community members older than 18 years	2009	Cross-sectional	1868	18-85	DSM-IV	HSCL	Central African Republic
Wenzel, T., F. Rushiti, et al. (2009). "Suicidal ideation, post-traumatic stress and suicide statistics in Kosovo. An analysis five years after the war. Suicidal ideation in Kosovo." Torture 19(3): 238-247.	Population over 15	2008	Cross-sectional	1161	15-8	DSM-IV	CIDI	Kosovo

Table S5: Summary of PTSD data sources

Full reference	Study population	Survey Year	Study design	Sample size	Age range	Diagnostic criteria	Measurement instrument	Country
Abbo, C., E. Kinyanda, et al. (2013). "Prevalence, comorbidity and predictors of anxiety disorders in children and adolescents in rural north-eastern Uganda." Child Adolesc Psychiatry Ment Health 7(1): 21.	General	2010	Cross-sectional	1587	3-19	DSM-IV	MINI-KID	Uganda
Acarturk, C., et al. (2017). "Prevalence and predictors of posttraumatic stress and depression symptoms among Syrian refugees in a refugee camp." Journal of Nervous and Mental Disease.	Adult resident, oldest in each house	2013	Cross-sectional	781	18-65	DSM-IV	IES-R	Syria
Alhasnawi S, Sadik S, Rasheed M, Baban A, et al. The prevalence and correlates of DSM-IV disorders in the Iraq Mental Health Survey (IMHS). World Psychiatry 2009;8:97-109	General	2007	Cross-sectional	4332	18-85	DSM-IV	CIDI	Iraq
Al-Jawadi, A. A. and S. Abdul-Rhman (2007). "Prevalence of childhood and early adolescence mental disorders among children attending primary health care centers in Mosul, Iraq: a cross-sectional study." BMC Public Health 7: 274.	Children of mothers presenting at PHC for vaccination	2004	Cross-sectional	3097	1-15	DSM-IV	DSM-IV checklist	Iraq
Ayazi, T., et al. (2012). "What are the risk factors for the comorbidity of posttraumatic stress disorder and depression in a war-affected population? A cross-sectional community study in South Sudan." BMC Psychiatry 12: 175	General population of four states	2010	Cross-sectional	1200	18-65	DSM-IV	HTQ	South Sudan
Banal, R., et al. (2010). "Psychiatric morbidity in adult Kashmiri migrants living in a migrant camp at Jammu." Indian Journal of Psychiatry 52(2): 154-158.	Parents of children in the camp	2010	Cross-sectional	600	15-65	DSM-IV	MINI	Kashmir
Ben-Ezra, M., et al. (2015). "Factors predicting risk for PTSD among highly exposed civilians: two longitudinal studies." Psychiatr Q 86(1): 19-32.	Adult household members	2009	Cross-sectional	194	18-85	DSM-IV	PCL-C	Israel
Benjet, C., G. Borges, et al. (2008). "Youth mental health in a populous city of the developing world: results from the	Adolescent residents of Mexico	2005	Cross-sectional	3005	12-17	DSM-IV	CIDI	Mexico

Mexican Adolescent Mental Health Survey." Journal of Child Psychology and Psychiatry 50(4): 486-495.	City Metropolitan Area								
Besser, A. and Y. Neria (2009). "PTSD symptoms, satisfaction with life, and prejudicial attitudes toward the adversary among Israeli civilians exposed to ongoing missile attacks." J Trauma Stress 22(4): 268-275.	Jewish adults	2008	Cross-sectional	341	20-60	DSM-IV	IES-R	Israel	
Bilanakis N, Pappas E, Bladic V, Jokic M. 1997. PTSD in a refugee camp in Serbia. Torture 7(1).	Refugees from Banja Luka, Bosnia-Herzegovina	1995	Cross-sectional	58	18-64	DSM-III	HTQ	Bosnia-Herzegovina	
Buljan D, Vrcek D, Cekic-Arambasin A, et al. Posttraumatic stress disorder, alcohol dependence, and somatic disorders in displaced persons. Alcoholism 2002; 48 (1-2): 45-40.	displaced persons, now placed in Vojnic, which provided accommodation for displaced persons from Banja Luka	2001	Cross-sectional	200	18-60	DSM-IV	SCID	Bosnia-Herzegovina	
Canetti, D., et al. (2010). "Exposure to prolonged socio-political conflict and the risk of PTSD and depression among Palestinians." Psychiatry 73(3): 219-231.	Adult residents	2007	Cross-sectional	138	18-85	DSM-IV	PSS	Israel	
Cardozo BL, Bilukha OO, Gotway CA, Wolfe MI. Mental Health of Women in Postwar Afghanistan. JOURNAL OF WOMEN'S HEALTH Volume 14, Number 4, 2005	Adult residents	2002	Cross-sectional	699	15-85	DSM-IV	HTQ	Afghanistan	
Cardozo BL, Kaiser, Gotway, Agani. Mental Health, Social Functioning, and Feelings of Hatred and Revenge of Kosovar Albanians One Year After the War in Kosovo. Journal of Traumatic Stress, Vol. 16, No. 4, August 2004, pp. 451–460.	Kosovar Albanians from 1991 census	2000	Cross-sectional	1399	15-85	DSM-IV	HTQ	Kosovo	

Cardozo BL, Vergara A, Agani F, Gotway CA. 2000. Mental health, social functioning, and attitudes of Kosovar Albanians following the War in Kosovo. JAMA 284;569-577.	Kosovar Albanians gfrom the 1991 census	1999	Cross-sectional	1358	15-85	DSM-IV	HTQ	Kosovo
Chipman, K. J., P. A. Palmieri, et al. (2011). "Predictors of posttraumatic stress-related impairment in victims of terrorism and ongoing conflict in Israel." Anxiety Stress Coping 24(4): 255-271.	Israeli telephone database	2008	Cross-sectional	1001	18-85	DSM-IV	PSS	Israel
de Jong K, Mulhern M, Ford N, et al. The trauma of war in Sierra Leone. THE LANCET • Vol 455 • June 10, 2000		1999	Cross-sectional	245	15-85	DSM-III	IES	Sierra Leone
Dymi, K., O. V. Rasmussen, et al. (2004). "Survey on frequency of types of trauma, and prevalence of PTSD symptomatology among Kosovo Albanian refugees, in the Tirana area refugee camps, in Albania, June 1999." Torture 14(Supplementum): 14-18.	Kosovo refugees in Albania	1999	Cross-sectional	158	15-70	DSM-IV	HTQ	Kosovo
Farhood L, Dimassi H, Lehtinen T. Exposure to War-Related Traumatic Events, Prevalence of PTSD, and General Psychiatric Morbidity in a Civilian Population From Southern Lebanon. J Transcult Nurs 2006 17: 444.		2001	Cross-sectional	256	20-85	DSM-III	HTQ	Lebanon
Farhood, L. F. and H. Dimassi (2011). "Prevalence and predictors for post-traumatic stress disorder, depression and general health in a population from six villages in South Lebanon." Soc Psychiatry Psychiatr Epidemiol.	Six villages >14 years old	2005	Cross-sectional	632	20-85	DSM-IV	HTQ	Lebanon
Farhood, L. F., et al. (2016). "PTSD and depression construct: Prevalence and predictors of co-occurrence in a South Lebanese civilian sample." European Journal of Psychotraumatology 7.	Adult residents	2007	Cross-sectional	758	20-85	DSM-IV	HTQ	Lebanon
Finkelstein, M. (2016). "Resource loss, resource gain, PTSD, and dissociation among Ethiopian immigrants in Israel." Scandinavian Journal of Psychology 57(4): 328-337.	Adult refugees	2001	Cross-sectional	144	18-85	DSM-IV	HTQ	Israel
Fox S, Tang SS. The Sierra Leonean Refugee Experience: Traumatic Events and Psychiatric Sequelae. The Journal of Nervous and Mental Disease Issue: Volume 188(8), August 2000, pp 490-495	Adult refugees maintaining regular contact	1998	Cross-sectional	55	18-70	DSM-III	HTQ	Sierra Leone

	with the Kerr Al-Hassan refugee camp							
Franciskovic T, Tovilovic Z, Sukovic Z, Stevanovic A, et al. 2008. Health care and community-based interventions for war-traumatised people in Croatia. Croat Med Journal 49:484-90.	Inhabitants from 3 Croatian counties directly exposed to war	1998	Cross-sectional	719	18-65	DSM-IV	MINI	Croatia
Galea, S., P. C. Rockers, et al. (2010). "Persistent psychopathology in the wake of civil war: long-term posttraumatic stress disorder in Nimba County, Liberia." Am J Public Health 100(9): 1745-1751.	Adults in national census data	2008	Cross-sectional	1376	19-85	DSM-IV	HTQ	Liberia
Gelkopf, M., et al. (2008). "The mental health impact of terrorism in Israel: a repeat cross-sectional study of Arabs and Jews." Acta Psychiatrica Scandinavica 117(5): 369-380.	Adult residents	2004	Cross-sectional	512	18-85	DSM-IV	SASRQ	Israel
Gelkopf, M., et al. (2013). "A longitudinal study of changes in psychological responses to continuous terrorism." Israel Journal of Psychiatry and Related Sciences 50(2): 100-109.	Adult residents	2004	Cross-sectional	153	18-85	DSM-IV	SASRQ	Israel
Good, M., B. Good, et al. (2007). "A psychosocial needs assessment of communities in 14 conflict-affected districts in Aceh." Banda Aceh: International Organization for Migration.	Households from a list compiled with assistance from subdistrict leaders and village heads	2006	Cross-sectional	1972	17-82	DSM-IV	HTQ	Indonesia
Halevi, G., et al. (2016). "Risk and resilience trajectories in war-exposed children across the first decade of life." Journal of Child Psychology and Psychiatry 57(10): 1183-1193.	Child residents	2008	Longitudinal	232	1-5	DSM-IV	DC 0-3R	Israel

Hall BJ, Hobfoll SE, Palmieri PA, et al. 2008. The psychological impact of impending forced settler disengagement in Gaza: trauma and post-traumatic growth. Journal of Traumatic Stress 21 (1): 22-29.	Israeli settlers in Gaza	2006	Cross-sectional	190	18-85	DSM-IV	PSS	Palestine
Hamid, A. A. and S. A. Musa (2010). "Mental health problems among internally displaced persons in Darfur." Int J Psychol 45(4): 278-285.	IDPs	2005	Cross-sectional	430	12-85	DSM-IV	PCL-C	Sudan
Hashemian F, Khoshnood K, Desai MM, et al. 2006. Anxiety, depression and PTSD in Iranian survivors of chemical warfare. JAMA 296:560-566	Civilians from Sardasht (high-intensity and chemical warfare)	2004	Cross-sectional	153	18-80	DSM	CAPS	Iran
Hobfoll, S. E., D. Canetti-Nisim, et al. (2008). "The association of exposure, risk, and resiliency factors with PTSD among Jews and Arabs exposed to repeated acts of terrorism in Israel." J Trauma Stress 21(1): 9-21.	Adult Israeli residents, Jewish	2004	Cross-sectional	1511	18-85	DSM-IV	PSS	Israel
Hobfoll, S. E., et al. (2011). "Are community studies of psychological trauma's impact accurate?: a study among Jews and Palestinians." Psychological Assessment 23(3): 599-605.	Israeli Jewish citizens	2007	Cross-sectional	75	18-85	DSM-IV	CIDI	Israel
Husain, F., M. Anderson, et al. (2011). "Prevalence of war-related mental health conditions and association with displacement status in postwar Jaffna District, Sri Lanka." Journal of the American Medical Association 406(5): 522-541.	Adult residents	2009	Cross-sectional	1409	15-85	DSM-IV	HTQ	Sri Lanka
Igreja, V., W. Kleijn, et al. (2006). "When the war was over, little changed: women's posttraumatic suffering after the war in Mozambique." Journal of Nervous and Mental Disease 194(7): 502-509.	All women in 2 villages	1998	Cross-sectional	91	22-60	DSM-IV	Self-rating Inventory for PTSD, modified	Mozambique
Johnson K, Asher J, Rosborough S, et al. 2008 Association of combatant status and sexual violence with health and mental health outcomes in postconflict Liberia. JAMA 400(6):676-690	Households from UNDP population	2008	Cross-sectional	1666	18-85	DSM-IV	PSS	Liberia

	information							
Johnson, K., J. Scott, et al. (2010). "Association of sexual violence and human rights violations with physical and mental health in territories of the eastern Democratic Republic of the Congo."	Adult household members from 4 districts	2010	Cross-sectional	998	18-85	DSM-IV	PSS	DRC
Karam EG. Comorbidity of posttraumatic stress disorder and depression. In Book Posttraumatic stress disorder 1997 American Psychiatric Press, Washington DC.	Individuals from four Lebanese communities differentially exposed to war.	1991	Cross-sectional	244	18-85	DSM-III	DIS	Lebanon
Karunakara UK, Neuner F, Chauer M, et al 2004. Traumatic events and symptoms of PTSD amongst Sudanese nationals, refugees and Ugandans in the West Nile. African Health Sciences 4(2).	Residents of sub-counties in Southern Sudan	2000	Cross-sectional	3323	18-85	DSM-IV	PDS, modified	Sudan, Uganda
Kashdan, T. B., et al. (2009). "Post-traumatic stress disorder, social anxiety disorder, and depression in survivors of the Kosovo War: experiential avoidance as a contributor to distress and quality of life." J Anxiety Disord 23(2): 185-196.	Adult residents	2006	Cross-sectional	174	18-85	DSM-IV	MINI	Kosovo
Klaric, M., T. Franciskovic, et al. (2008). "Social support and PTSD symptoms in war-traumatized women in Bosnia and Herzegovina." Psychiatr Danub 20(4): 466-474.	Adult women	2004	Cross-sectional	187	28-65	DSM-IV	HTQ	Bosnia-Herzegovina
Kohrt, B. A., D. J. Hruschka, et al. (2012). "Political violence and mental health in Nepal: prospective study*." The British Journal of Psychiatry.	Adults in Jumla	2007	Longitudinal	298	18-85	DSM-IV	PCL-C	Nepal
Kozaric-Kovacic D, Ljubin T, Grappe M. 2000 Comorbidity of PTSD and alcohol dependence in displaced persons. Croatian Med Journal 41(2): 174-178.	Adult Croatian displaced persons in camps	1997	Cross-sectional	368	18-77	DSM-III	SCID	Croatia

	near Zagreb							
Lecic-Tosevski, D., et al. (2013). "Posttraumatic stress disorder in a Serbian community: seven years after trauma exposure." J Nerv Ment Dis 201(12): 1040-1044.	Adult residents	2006	Cross-sectional	640	18-85	DSM-IV	MINI	Serbia
Lopes Cardozo, B., et al. (2004). "Karenni refugees living in Thai-Burmese border camps: traumatic experiences, mental health outcomes, and social functioning." Social Science and Medicine 58(12): 2637-2644.	Adult refugees	2001	Cross-sectional	495	15-85	DSM-IV	HTQ	Myanmar
Luitel, N. P., M. J. Jordans, et al. (2013). "Conflict and mental health: a cross-sectional epidemiological study in Nepal." Soc Psychiatry Psychiatr Epidemiol 38(2): 183-193.	Adult residents	2008	Cross-sectional	720	18-85	DSM-IV	PCL-C	Nepal
Madianos, M. G., et al. (2011). "Posttraumatic stress disorders comorbid with major depression in west bank, palestine: A general population cross sectional study." European Journal of Psychiatry 25(1): 19-31.	Palestinians in four areas of West Bank	2007	Cross-sectional	916	18-85	DSM-IV	SCID	Palestine
Makhashvili, N., et al. (2014). "Mental disorders and their association with disability among internally displaced persons and returnees in Georgia." Journal of Traumatic Stress 27(5): 509-518.	Adult residents	2011	Cross-sectional	3025	18-85	DSM-IV	TSQ	Georgia
Marušić, A., D. Kozarić-Kovačić, et al. (1995). "Use of two PTSD scales in assessing posttraumatic stress disorder in refugees and displaced persons from Bosnia and Herzegovina and Croatia." Psychologische Beitrage 47(1-2): 209-214.	List of refugee camp inhabitants	1994	Cross-sectional	73	18-85	DSM-III	PTSD-I	Croatia
Modvig J, Pagaduan-Lopez J, Rodenburg J, Salud CMD, Cabigon RV, et al. (2000) Torture and trauma in post-conflict East Timor. The Lancet 456: 1764.	1033 households in 13 districts of East Timor	2000	Cross-sectional	1033	12-80	DSM	HTQ	Timor-Leste
Mollica, R. F., et al. (2001). "Longitudinal study of psychiatric symptoms, disability, mortality, and emigration among Bosnian refugees." Journal of the American Medical Association 286(5): 546-554.	Bosnian refugees	1999	Cross-sectional	114	18-85	DSM-IV	HTQ	Bosnia-Herzegovina
Mollica, R. F., et al. (2002). "Science-based policy for psychosocial interventions in refugee camps: a Cambodian	Adult refugees	1988	Cross-sectional	993	18-85	DSM-III	HTQ	Cambodia

example." Journal of Nervous and Mental Disease 190(3): 158-166.								
Morina, N. and J. D. Ford (2008). "Complex sequelae of psychological trauma among Kosovar civilian war victims." Int J Soc Psychiatry 54(5): 425-436.	Adult residents	2005	Cross-sectional	102	18-65	DSM-IV	MINI	Kosovo
Mufti, K. A., F. Naeem, et al. (2005). "Psychiatric problems in an Afghan village." J Ayub Med Coll Abbottabad 17(4): 19-20.	Inhabitants >16 of small village	2004	Cross-sectional	1301	15-70	DSM-IV	MINI	Afghanistan
Mugisha, J., et al. (2015). "Prevalence and factors associated with Posttraumatic Stress Disorder seven years after the conflict in three districts in northern Uganda (The Wayo-Nero Study)." BMC Psychiatry 15: 170.	Adult residents	2013	Cross-sectional	2361	18-65	DSM-IV	MINI	Uganda
Munyandamutsa, N., P. Mahoro Nkubamugisha, et al. (2012). "Mental and physical health in Rwanda 14 years after the genocide." Soc Psychiatry Psychiatr Epidemiol.	Adult residents	2008	Cross-sectional	962	16-85	DSM-IV	MINI	Rwanda
Neugebauer, R., P. W. Fisher, et al. (2009). "Post-traumatic stress reactions among Rwandan children and adolescents in the early aftermath of genocide." Int J Epidemiol 48(4): 1044-1045.	8-19 years old living in the community	1995	Cross-sectional	942	8-19	DSM-IV	IES, modified	Rwanda
Ng, L. C., et al. (2017). "Posttraumatic stress disorder, trauma, and reconciliation in South Sudan." Social Psychiatry and Psychiatric Epidemiology.	Adult residents	2015	Cross-sectional	1520	18-85	DSM-IV	HTQ	South Sudan
Nuttman-Shwartz, O., et al. (2015). "Continuous exposure to life threats among different age groups in different types of communities." Psychol Trauma 7(3): 269-276.	Adult residents	2009	Cross-sectional	276	20-75	DSM-IV	PTSD Inventory	Israel
Onyut LP, Neuner F, Ertl V, et al. Trauma, poverty and mental health among Somali and Rwandese refugees living in an African refugee settlement – an epidemiological study. Conflict and Health 2009, 4:6	All Rwandese (Hutu) refugees of either sex above the age of 12 officially	2003	Cross-sectional	906	12-85	DSM-IV	PDS	Somalia

	registered and resident							
Palgi, Y. (2017). "Matter of will: the association between posttraumatic stress symptoms and the will-to-live." Psychiatry Research 249: 180-186.	Adult residents over age 50	2014	Cross-sectional	170	50-85	DSM-V	PCL	Israel
Palmieri, P. A., D. Canetti-Nisim, et al. (2008). "The psychological impact of the Israel-Hezbollah War on Jews and Arabs in Israel: the impact of risk and resilience factors." Soc Sci Med 67(8): 1208-1216.	>18 years from telephone directory	2006	Cross-sectional	1200	18-85	DSM-IV	PSS	Israel
Palmieri, P. A., et al. (2010). "Prevalence and correlates of sleep problems in adult Israeli Jews exposed to actual or threatened terrorist or rocket attacks." J Clin Sleep Med 6(6): 557-564.	Adult residents	2008	Cross-sectional	500	18-85	DSM-IV	PSS	Israel
Pedersen, D., J. Tremblay, et al. (2008). "The sequelae of political violence: assessing trauma, suffering and dislocation in the Peruvian highlands." Soc Sci Med 67(2): 205-217.	Adults of households	2002	Cross-sectional	373	14-85	DSM-III	HTQ, modified	Peru
Peltzer, K. (1999). "Posttraumatic stress symptoms in a population of rural children in South Africa." Psychological Reports 85(2): 646-650.	All children in community	1997	Cross-sectional	148	6-16	DSM-III	CPTSDI	South Africa
Pham, P. N., H. M. Weinstein, et al. (2004). "Trauma and PTSD symptoms in Rwanda: implications for attitudes toward justice and reconciliation." JAMA 292(5): 602-612.		2002	Cross-sectional	2091	18-85	DSM-IV	PCL-C	Rwanda
Priebe, S., et al. (2010). "Mental disorders following war in the Balkans: a study in 5 countries." Arch Gen Psychiatry 67(5): 518-528.	Adult residents	2005	Cross-sectional	2652	20-65	DSM-IV	MINI	Bosnia-Herzegovina, Croatia, Kosovo, Serbia
Qouta, S. and J. Odeb (2005). "The impact of conflict on children: the Palestinian experience." J Ambul Care Manage 28(1): 75-79.	Children across Gaza Strip	2003	Cross-sectional	944	10-19	DSM-IV	CPTSD-RI	Palestine

Rasekh, Z., H. M. Bauer, et al. (1998). "Women's health and human rights in Afghanistan." JAMA 280(5): 449-455.	Female heads of households NGO lists	1998	Cross-sectional	160	17-55	DSM-IV	DSM-IV checklist	Afghanistan
Rieder, H. and T. Elbert (2013). "Rwanda -- lasting imprints of a genocide: trauma, mental health and psychosocial conditions in survivors, former prisoners and their children." Confl Health 7(1): 6.	Population of Muhanga district	2010	Cross-sectional	90	30-77	DSM-IV	PSS	Rwanda
Roberts B, Damunda EY, Lomoro O, Sondorp E. Post-conflict mental health needs: a cross-sectional survey of trauma, depression and associated factors in Juba, Southern Sudan. BMC Psychiatry 2009, 9:7.		2007	Cross-sectional	1242	18-85	DSM-IV	HTQ	Sudan
Roberts, B., et al. (2008). "The reliability and validity of the SF-8 with a conflict-affected population in northern Uganda." Health and Quality of Life Outcomes 6.	Adult IDPs	2006	Cross-sectional	1206	18-84	DSM-IV	HTQ	Uganda
Rosner R, Powell S, Butollo W. Posttraumatic Stress Disorder Three Years After the Siege of Sarajevo. JOURNAL OF CLINICAL PSYCHOLOGY, Vol. 59(1), 41–55 (2004)	Population of Sarajevo	1998	Cross-sectional	98	16-65	DSM-IV	PDS, modified	Bosnia-Herzegovina
Salah, T. T., et al. (2013). "The mental health of internally displaced persons: an epidemiological study of adults in two settlements in Central Sudan." The International journal of social psychiatry 59(8): 782-788.	Adult IDPs	2008	Cross-sectional survey	1876	18-85	DSM-IV	MINI	South Sudan
Schaal, S., et al. (2012). "Mental health 15 years after the killings in Rwanda: Imprisoned perpetrators of the genocide against the Tutsi versus a community sample of survivors." Journal of Traumatic Stress 25(4): 446-453.	Adult survivors of genocide	2009	Cross-sectional survey	114	18-85	DSM-IV	PSS	Rwanda
Schick, M., et al. (2013). "Trauma, mental health, and intergenerational associations in Kosovar families 11 years after the war." European Journal of Psychotraumatology 4.	Child residents and their parents	2010	Cross-sectional survey	153	10-65	DSM-IV	PDS	Kosovo
Scholte, W. F., et al. (2004). "Mental health symptoms following war and repression in eastern Afghanistan." JAMA 292(5): 585-593.	Adult residents	2003	Cross-sectional survey	1011	15-85	DSM-IV	HTQ	Afghanistan
Shalev AY, Tuval R, Frenkiel-Fishman S, Hadara H, Eth S. Psychological Responses to Continuous Terror: A Study of	Community members listed in	2001	Cross-sectional	256	18-85	DSM-IV	PSS	Israel

Two Communities in Israel. Am J Psychiatry 2006; 164:667–674	the telephone directory							
Sheikh, T. L., et al. (2015). Correlates of depression among internally displaced persons after post-election violence in Kaduna, North Western Nigeria. Netherlands, Elsevier Science. 170: 46-51.	Adult IDPs	2013	Cross-sectional	258	18-85	DSM-IV	HTQ	Nigeria
Silove, D., C. R. Bateman, et al. (2008). "Estimating clinically relevant mental disorders in a rural and an urban setting in postconflict Timor Leste." Arch Gen Psychiatry 65(10): 1205-1212.	Adult residents of a rural and urban area	2006	Cross-sectional	1544	18-85	DSM-IV	SCID	Timor-Leste
Somer, E., A. Zrihan-Weitzman, et al. (2009). "Israeli civilians under heavy bombardment: prediction of the severity of post-traumatic symptoms." Prehosp Disaster Med 24(5): 489-494.	Hebrew speaking adults from the telephone directory	2006	Cross-sectional	317	18-85	DSM-IV	PSS	Israel
Stammel, N., et al. (2015). "Applicability of the ICD-11 proposal for PTSD: A comparison of prevalence and comorbidity rates with the DSM-IV PTSD classification in two post-conflict samples." European Journal of Psychotraumatology 6.	Adult IDPs	2012	Cross-sectional	453	18-85	DSM-IV	PCL-C	Colombia
Tang, S. S. and S. H. Fox (2001). "Traumatic experiences and the mental health of Senegalese refugees." J Nerv Ment Dis 189(8): 507-512.	Adult refugees	2001	Cross-sectional	80	18-85	DSM-III	HTQ	Senegal
Tekin, A., et al. (2016). "Prevalence and gender differences in symptomatology of posttraumatic stress disorder and depression among Iraqi Yazidis displaced into Turkey." European Journal of Psychotraumatology 7.	Adult refugees	2015	Cross-sectional	238	18-65	DSM-IV	SCID	Iraq
Thabet, A. A. M., A. A. Tawahina, et al. (2007). "Children exposed to political conflict: implications for health policy." Harvard Health Policy Review 8(2): 144-154.	Child residents	2004	Cross-sectional	409	9-18	DSM-IV	CRIES	Palestine
Thabet, A. A., et al. (2008). "Exposure to war trauma and PTSD among parents and children in the Gaza strip." Eur Child Adolesc Psychiatry 17(4): 191-199.	Parents in families with	2006	Cross-sectional	200	9-65	DSM-IV	PCL	Palestine

	mother, father, son, and daughter							
Thabet, A. A., Y. Abed, et al. (2001). "Effect of trauma on the mental health of Palestinian children and mothers in the Gaza Strip." East Mediterr Health J 7(4): 414-421.		2000	Cross-sectional	286	9-18	DSM-III	IES	Palestine
Thapa, S. B. and E. Hauff (2005). "Psychological distress among displaced persons during an armed conflict in Nepal." Soc Psychiatry Psychiatr Epidemiol 40(8): 672-679.	Adult IDPs	2003	Longitudinal	290	18-79	DSM-IV	PCL-C	Nepal
Tracy, M., S. E. Hobfoll, et al. (2008). "Predictors of depressive symptoms among Israeli Jews and Arabs during the Al aqsa intifada: a population-based cohort study." Ann Epidemiol 18(6): 447-457.	Israeli telephone company lists	2004	Longitudinal	1613	18-85	DSM-IV	PSS	Israel
Tremblay, J., D. Pedersen, et al. (2009). "Assessing mental health outcomes of political violence and civil unrest in Peru." Int J Soc Psychiatry 55(5): 449-464.	Adults of 5 communities	2001	Cross-sectional	373	15-85	DSM-III	HTQ, modified	Peru
Vinck P, Pham P, Stover E, Weinstein HM. Exposure to War Crimes and Implications for Peace Building in Northern Uganda. JAMA, August 1, 2007—Vol 298, No. 5		2005	Cross-sectional	2585	18-85	DSM-IV	PCL-C	Uganda
Vinck, P. and N. Phuong (2012). "Association of Exposure to Intimate-partner Physical Violence and Potentially Traumatic War-Related Events with Mental Health in Liberia." Social Science & Medicine.	2008 census	2010	Cross-sectional	4501	18-85	DSM-IV	PCL-C	Liberia
Wang, S. J., F. Rushiti, et al. (2012). "Survivors of war in northern Kosovo (III): The role of anger and hatred in pain and PTSD and their interactive effects on career outcome, quality of sleep and suicide ideation." Confl Health 6(1): 4.	Victims of violence, torture, rape, arrest or execution of family members	2008	Cross-sectional	125	18-85	DSM-IV	HTQ	Kosovo
Wenzel, T., F. Rushiti, et al. (2009). "Suicidal ideation, post-traumatic stress and suicide statistics in Kosovo. An analysis five years after the war. Suicidal ideation in Kosovo." Torture 19(4): 248-247.	Population over 15	2008	Cross-sectional	1161	15-85	DSM-IV	HTQ	Kosovo

Yasan A, Saka G, Ozkan M, Ertem M. Trauma Type, Gender, and Risk of PTSD in a Region Within an Area of Conflict. Journal of Traumatic Stress, Vol. 22, No. 6, December 2009, pp. 664–666	Inhabitants of Diyarbakir	2005	Cross-sectional	708	18-85	DSM-IV	CAPS	Turkey

Table S6: Summary of anxiety data sources

Full reference	Study population	Survey Year	Study design	Sample size	Age range	Diagnostic criteria	Measurement instrument	Country
Abbo C, Kinyanda E, Kizza RB, Levin J, Ndyanabangi S, Stein DJ. Prevalence, comorbidity and predictors of anxiety disorders in children and adolescents in rural north-eastern Uganda. Child Adolesc Psychiatry Ment Health. 2013; 21.	General population	2010	Cross-sectional	1587	3-19	DSM-IV	MINI-KID	Uganda
Alhasnawi S, SADIK S, RASHEED M, BABAN A, AL-ALAK MM, OTHMAN AY, et al. The prevalence and correlates of DSM-IV disorders in the Iraq Mental Health Survey (IMHS). World Psychiatry. 2009;8(2):97.	General population	2006	Cross-sectional	4332	18-85	DSM-IV	CIDI	Iraq
Al-Jawadi AA, Abdul-Rhman S. Prevalence of childhood and early adolescence mental disorders among children attending primary health care centers in Mosul, Iraq: a cross-sectional study. BMC Public Health. 2007; 7: 274.	Primary healthcare	2003	Cross-sectional	3097	1-15	DSM-IV	DSM-IV checklist	Iraq
Banal, R., et al. (2010). "Psychiatric morbidity in adult Kashmiri migrants living in a migrant camp at Jammu." Indian Journal of Psychiatry 52(2): 154-158.	Parents of children in the camp	2010	Cross-sectional	600	15-65	DSM-IV	MINI	Kashmir
Benjet, C., G. Borges, et al. (2008). "Youth mental health in a populous city of the developing world: results from the Mexican Adolescent Mental Health Survey." Journal of Child Psychology and Psychiatry 50(4): 486-495.	Adolescent residents of Mexico City Metropolitan Area	2005	Cross-sectional	3005	12-17	DSM-IV	CIDI	Mexico
Cardozo BL, Bilukha OO, Gotway CA, Wolfe MI. Mental Health of Women in Postwar Afghanistan. JOURNAL OF WOMEN'S HEALTH Volume 14, Number 4, 2005.	Adult residents	2002	Cross-sectional	699	15-85	DSM-IV	HSCL	Afghanistan
Farbstein I, Mansbach-Kleinfeld I, Levinson D, Goodman R, Levav I, Vograft I, et al. Prevalence and correlates of mental disorders in Israeli adolescents: results from a national mental health survey. Journal of Child Psychology and Psychiatry. 2010;51(5):630-9.	General population	2010	Cross-sectional		14-17	DSM-IV	DAWBA	Israel
Fox S, Tang SS. The Sierra Leonean Refugee Experience: Traumatic Events and Psychiatric Sequelae. The Journal of	Adult refugees maintainin	1998	Cross-sectional	55	18-70	DSM-III	HSCL	Sierra Leone

Nervous and Mental Disease Issue: Volume 188(8), August 2000, pp 490-495	g regular contact with the Kerr Al-Hassan refugee camp							
Husain, F., M. Anderson, et al. (2011). "Prevalence of war-related mental health conditions and association with displacement status in postwar Jaffna District, Sri Lanka." Journal of the American Medical Association 406(5): 522-541.	Adult residents	2009	Cross-sectional	1409	15-85	DSM-IV	HSCL	Sri Lanka
Karam EG, Mneimneh ZN, Karam AN, Fayyad JA, Nasser SC, Chatterji S, et al. Prevalence and treatment of mental disorders in Lebanon: A national epidemiological survey. The Lancet. 2006;367(9515):1000-6.	General population	2003	Cross-sectional	1031	18-85	DSM-IV	CIDI	Lebanon
Karam, G., Itani, L., Fayyad, J., Karam, A., Mneimneh, Z., & Karam, E. (2016). Prevalence, correlates, and treatment of mental disorders among Lebanese older adults: a national study. The American Journal of Geriatric Psychiatry, 24(4), 278-286.	General population	2003	Cross-sectional	2857	60-99	DSM-IV	CIDI	Lebanon
Khaled N. Psychological effects of terrorist attacks in Algeria. Journal of Aggression, Maltreatment and Trauma. 2004;9(1):201-12.	General population	1999	Cross-sectional	652	15-19	DSM-IV	CIDI	Algeria
Kohrt, B. A., D. J. Hruschka, et al. (2012). "Political violence and mental health in Nepal: prospective study*." The British Journal of Psychiatry.	Adults in Jumla	2007	Longitudinal	298	18-85	DSM-IV	BAI	Nepal
Kohrt, B. A., Speckman, R. A., Kunz, R. D., Baldwin, J. L., Upadhaya, N., Acharya, N. R., ... & Worthman, C. M. (2009). Culture in psychiatric epidemiology: using ethnography and multiple mediator models to assess the relationship of caste with depression and anxiety in Nepal. Annals of human biology, 36(3), 261-280.	Adult residents of Jumla	2000	Cross-sectional	307	18-85	DSM-IV	BAI	Nepal
Llosa AE, Ghantous Z, Souza R, Forgione F, Bastin P, Jones A, et al. Mental disorders, disability and treatment gap in a protracted refugee setting. The British Journal of Psychiatry. 2014;204(3):208-13.	General population	2010	Cross-sectional			DSM-IV	MINI	Lebanon

Lopes Cardozo, B., et al. (2004). "Karenni refugees living in Thai-Burmese border camps: traumatic experiences, mental health outcomes, and social functioning." Social Science and Medicine 58(12): 2637-2644.	Adult refugees	2001	Cross-sectional	495	15-85	DSM-IV	HSCL	Myanmar
Luitel, N. P., M. J. Jordans, et al. (2013). "Conflict and mental health: a cross-sectional epidemiological study in Nepal." Soc Psychiatry Psychiatr Epidemiol 38(2): 183-193.	Adult residents	2008	Cross-sectional	720	18-85	DSM-IV	BAI	Nepal
Maalouf, F. T., et al. (2016). "Psychiatric disorders among adolescents from Lebanon: Prevalence, correlates, and treatment gap." Social Psychiatry and Psychiatric Epidemiology 51(8): 1105-1116.	Adolescent residents of Beirut	2012	Cross-sectional	510	11-17	DSM-IV	DAWBA	Lebanon
Makhashvili, N., et al. (2014). "Mental disorders and their association with disability among internally displaced persons and returnees in Georgia." Journal of Traumatic Stress 27(5): 509-518.	Adult residents	2011	Cross-sectional	3025	18-85	DSM-IV	GAD-7	Georgia
McConnell, P., P. Bebbington, et al. (2002). "Prevalence of psychiatric disorder and the need for psychiatric care in northern Ireland: Population study in the district of Derry." British Journal of Psychiatry 181(3): 214-219.	Electoral roll	1993	Cross-sectional	307	18-64	ICD-10	SCAN	Northern Ireland
Medina-Mora ME, Borges G, Lara C, Benjet C, Blanco J, Fleiz C, Villatoro J, Rojas E, Zambrano J: Prevalence, service use, and demographic correlates of 12-month DSM-IV psychiatric disorders in Mexico: Results from the Mexican National Comorbidity Survey. Psychological Medicine, 2005, 35(12):1773-83.	Adults in the Mexican National Comorbidity Survey	2002	Cross-sectional	5826	18-65	DSM-IV	CIDI	Mexico
Mufti, K. A., F. Naeem, et al. (2005). "Psychiatric problems in an Afghan village." J Ayub Med Coll Abbottabad 17(3): 19-20.	Inhabitants under age 16 years in a small village	2004	Cross-sectional	1301	15-70	DSM-IV	MINI	Afghanistan
Mugisha, J., et al. (2015). "Prevalence and factors associated with Posttraumatic Stress Disorder seven years after the conflict in three districts in northern Uganda (The Wayo-Nero Study)." BMC Psychiatry 15: 170.	Adult residents	2013	Cross-sectional	2361	18-65	DSM-IV	MINI	Uganda

MUMFORD, D. B., F. A. MINHAS, et al. (2000). "Stress and psychiatric disorder in urban Rawalpindi Community survey." The British Journal of Psychiatry 177(6): 557-562.	Adult residents	1994	Cross-sectional	774	18-80	ICD-10	Clinical diagnosis	Pakistan
Priebe, S., et al. (2010). "Mental disorders following war in the Balkans: a study in 5 countries." Arch Gen Psychiatry 67(5): 518-528.	Adult residents	2005	Cross-sectional	2652	20-65	DSM-IV	MINI	Bosnia-Herzegovina, Croatia, Kosovo, Serbia
Rieder, H. and T. Elbert (2013). "Rwanda -- lasting imprints of a genocide: trauma, mental health and psychosocial conditions in survivors, former prisoners and their children." Confl Health 7(1): 6.	Population of Muhanga district	2010	Cross-sectional	90	30-77	DSM-IV	HSCL	Rwanda
Salah, T. T., et al. (2013). "The mental health of internally displaced persons: an epidemiological study of adults in two settlements in Central Sudan." The International journal of social psychiatry 59(8): 782-788.	Adult refugees	2008	Cross-sectional	1876	18-85	DSM-IV	MINI	South Sudan
Schaal, S., et al. (2012). "Mental health 15 years after the killings in Rwanda: Imprisoned perpetrators of the genocide against the Tutsi versus a community sample of survivors." Journal of Traumatic Stress 25(4): 446-453.	Adult survivors of genocide	2009	Cross-sectional	114	18-85	DSM-IV	HSCL	Rwanda
Scholte, W. F., et al. (2004). "Mental health symptoms following war and repression in eastern Afghanistan." JAMA 292(5): 585-593.	Adult residents	2003	Cross-sectional	1011	15-85	DSM-IV	HSCL	Afghanistan
Stammel, N., et al. (2015). "Applicability of the ICD-11 proposal for PTSD: A comparison of prevalence and comorbidity rates with the DSM-IV PTSD classification in two post-conflict samples." European Journal of Psychotraumatology 6.	Adult IDPs	2012	Cross-sectional	453	18-85	DSM-IV	HSCL	Colombia
Tang, S. S. and S. H. Fox (2001). "Traumatic experiences and the mental health of Senegalese refugees." J Nerv Ment Dis 189(8): 507-512.	Adult refugees	2001	Cross-sectional	80	18-85	DSM-III	HSCL	Senegal
Thabet, A. A. M., A. A. Tawahina, et al. (2007). "Children exposed to political conflict: implications for health policy." Harvard Health Policy Review 8(2): 144-154.	Child residents	2004	Cross-sectional	409	9-18	DSM-IV	RCMAS	Palestine

Thabet, A. A., et al. (2008). "Exposure to war trauma and PTSD among parents and children in the Gaza strip." Eur Child Adolesc Psychiatry 17(4): 191-199.	Parents in families with mother, father, son, and daughter	2006	Cross-sectional	200	18-65	DSM-IV	MAS	Palestine
Thapa, S. B. and E. Hauff (2005). "Psychological distress among displaced persons during an armed conflict in Nepal." Soc Psychiatry Psychiatr Epidemiol 40(8): 672-679.	Adult IDPs	2003	Cross-sectional	290	18-79	DSM-IV	HSCL	Nepal
Vinck, P. and P. N. Pham (2010). "Association of exposure to violence and potential traumatic events with self-reported physical and mental health status in the Central African Republic." JAMA: Journal of the American Medical Association 304(5): 544-552.	Community members older than 18 years	2009	Cross-sectional	1868	18-85	DSM-IV	HSCL	Central African Republic
Vinck, P. and P. N. Pham (2013). "Association of exposure to intimate-partner physical violence and potentially traumatic war-related events with mental health in Liberia." Soc Sci Med 77: 41-49.	Adults randomly selected from 2008 census	2010	Cross-sectional	4501	18-85	DSM-IV	HSCL	Liberia
Wenzel, T., F. Rushiti, et al. (2009). "Suicidal ideation, post-traumatic stress and suicide statistics in Kosovo. An analysis five years after the war. Suicidal ideation in Kosovo." Torture 19(3): 238-247.	Population over 15 years of age	2008	Cross-sectional	1161	15-85	DSM-IV	HSCL	Kosovo
World Health Organization (WHO). WHO World Mental Health Surveys Report 2008. Geneva, Switzerland: World Health Organization (WHO), 2008.	General population	2003	Cross-sectional	4015	18-85	DSM-IV	CIDI	Colombia

Table S7: Relative risks of covariates estimated from DisMod-MR modelling

PTSD	RR (95%UI)	Depression	RR (95%UI)	Anxiety	RR (95%UI)
**Instrument type**					
Diagnostic interview	1.0	Diagnostic interview	1.0	Diagnostic interview	1.0
Symptom scale	1.5 (1.2-1.9)*	Symptom scale	1.8 (1.4-2.3)*	Symptom scale	1.9 (1.4-2.6)*
**Sex**					
Females	1.0	Females	1.0	Females	1.0
Males	(0.6-1.1)	Males	0.7 (0.5-0.9)*	Males	0.4 (0.7-1.0)
**Time since conflict**					
0 (still in conflict)	1.0	0 (still in conflict)	1.0	0 (still in conflict)	1.0
1-2 years	0.8 (0.6-1.0)	1-2 years	1.0 (0.7-1.3)	1-2 years	0.4 (0.3-0.7)*
3-4 years	1.0 (0.6-1.6)	3-4 years	0.6 (0.4-0.8)*	3-4 years	0.3 (0.2-0.5)*
5-7 years	1.0 (0.8-1.4)	5-7 years	1.2 (0.8-1.9)	5-7 years	1.2 (0.8-2.0)
8-10 years	1.0 (0.7-1.4)	8-10 years	1.2 (0.7-1.9)	8-10 years	1.4 (0.9-2.3)
**Response rate**					
>80%	1.0	>80%	1.0		
50-79%	0.5 (0.4-0.8)*	<80%	0.7 (0.5-1.2)		
**War-related events ratio**					
<0.3	1.0				
>0.3	1.0 (0.8-1.2)				
**Coverage**					
National	1.0				
Regional	1.4 (1.0-1.9)*				
Community	1.9 (1.3-2.5)*				

*Statistically significant finding

References

1. Uppsala Conflict Data Program. UCDP/PRIO Armed Conflict Dataset. 2017. http://www.prio.no/Data/Armed-Conflict/UCDP-PRIO/ (accessed 6 July 2017.
2. Sarkees M. R. The COW Typology of War: Defining and Categorizing Wars (Version 4 of the data). 2010. http://www.correlatesofwar.org/ (accessed 13/02/2013.
3. Integrated Network for Societal Conflict Research. Major Episodes of Political Violence (MEPV) and conflict regions, 1946-2008. 2012. http://www.systemicpeace.org/inscr/inscr.htm (accessed 13/02/2013.
4. Heidelberg Institute for International Conflict Research. Conflict barometer 2012: disputes - non-violent crises - violent crises - limited wars - wars. 2013. http://www.hiik.de/de/konfliktbarometer/pdf/ConflictBarometer_2012.pdf
5. Political Terror Scale. The Political Terror Scale. 2017. http://www.politicalterrorscale.org/Data/Data-Archive.html (accessed 6 July 2017.
6. Sim J, Wright CC. The kappa statistic in reliability studies: use, interpretation, and sample size requirements. *Physical therapy* 2005; **85**(3): 257-68.
7. Cohen J. A coefficient of agreement for nominal scales. *Educational and psychological measurement* 1960; **20**(1): 37-46.
8. Blanchard EB, Jones-Alexander J, Buckley TC, Forneris CA. Psychometric properties of the PTSD Checklist (PCL). *Behaviour Research and Therapy* 1996; **34**(8): 669-73.
9. Mollica RF, Caspi-Yavin Y, Bollini P, Truong T. The Harvard Trauma Questionnaire: validating a cross-cultural instrument for measuring torture, trauma, and posttraumatic stress disorder in Indochinese refugees. *Journal of Nervous and Mental Disease* 1992.
10. Derogatis LR, Lipman RS, Rickels K, Uhlenhuth EH, Covi L. The Hopkins Symptom Checklist (HSCL): A self-report symptom inventory. *Systems Research and Behavioral Science* 1974; **19**(1): 1-15.
11. Kessler RC, Üstün TB. The world mental health (WMH) survey initiative version of the world health organization (WHO) composite international diagnostic interview (CIDI). *International journal of methods in psychiatric research* 2004; **13**(2): 93-121.
12. Spitzer R, Williams J, Gibbon M, First M. The Structural Clinical Interview for DSM-III-R (SCID): 1. History, rationale, and description. *Arch Gen Psych* 1992; **49**: 624 - 9.
13. Blake DD, Weathers FW, Nagy LM, et al. The development of a clinician-administered PTSD scale. *Journal of Traumatic Stress* 1995; **8**(1): 75-90.
14. Robins LN, Helzer JE, Croughan J, Ratcliff KS. National Institute of Mental Health diagnostic interview schedule: its history, characteristics, and validity. *Archives of General Psychiatry* 1981; **38**(4): 381.
15. Foa EB, Riggs DS, Dancu CV, Rothbaum BO. Reliability and validity of a brief instrument for assessing post-traumatic stress disorder. *Journal of Traumatic Stress* 1993; **6**(4): 459-73.
16. Dozois DJ, Dobson KS, Ahnberg JL. A psychometric evaluation of the Beck Depression Inventory–II. *Psychological assessment* 1998; **10**(2): 83.
17. Sheehan DV, Lecrubier Y, Sheehan KH, et al. The Mini-International Neuropsychiatric Interview (M.I.N.I.): the development and validation of a structured diagnostic psychiatric interview for DSM-IV and ICD-10. *The Journal of clinical psychiatry* 1998; **59 Suppl 20**: 22-33;quiz 4-57.
18. Kroenke K, Spitzer RL. The PHQ-9: a new depression diagnostic and severity measure. *Psychiatric annals* 2002; **32**(9): 509-15.
19. Endicott J, Spitzer RL. A diagnostic interview: the schedule for affective disorders and schizophrenia. *Arch Gen Psychiatry* 1978; **35**(7): 837-44.
20. Wing JK, Babor T, Brugha T, et al. SCAN. Schedules for Clinical Assessment in Neuropsychiatry. *Arch Gen Psychiatry* 1990; **47**(6): 589-93.
21. Horowitz M, Wilner N, Alvarez W. Impact of Event Scale: a measure of subjective stress. *Psychosomatic medicine* 1979; **41**(3): 209-18.
22. Weiss DS. The impact of event scale: revised. Cross-cultural assessment of psychological trauma and PTSD: Springer; 2007: 219-38.

23. Cardena E, Koopman C, Classen C, Waelde LC, Spiegel D. Psychometric properties of the Stanford Acute Stress Reaction Questionnaire (SASRQ): A valid and reliable measure of acute stress. *Journal of traumatic stress* 2000; **13**(4): 719-34.
24. Zero To Three. Diagnostic classification of mental health and developmental disorders of infancy and early childhood: Revised edition (DC: 0-3R). *Zero To Three Press, Washington, DC Google Scholar* 1994.
25. Hovens J, Ploeg Hvd, Bramsen I, Klaarenbeek M, Schreuder J, Rivero VV. The development of the self-rating inventory for posttraumatic stress disorder. *Acta Psychiatrica Scandinavica* 1994; **90**(3): 172-83.
26. Walters JTR, Bisson JI, Shepherd JP. Predicting post-traumatic stress disorder: validation of the Trauma Screening Questionnaire in victims of assault. *Psychological Medicine* 2007; **37**(1): 143-50.
27. Watson CG, Juba MP, Manifold V, Kucala T, Anderson PE. The PTSD interview: Rationale, description, reliability, and concurrent validity of a DSM-III-based technique. *Journal of Clinical Psychology* 1991; **47**(2): 179-88.
28. Frederick C, Pynoos R, Nader K. Child post-traumatic stress disorder reaction index (CPTSD-RI). *Department of Psychiatry, University of California at Los Angeles, Los Angeles* 1992.
29. Perrin S, Meiser-Stedman R, Smith P. The Children's Revised Impact of Event Scale (CRIES): Validity as a screening instrument for PTSD. *Behavioural and Cognitive Psychotherapy* 2005; **33**(4): 487.
30. Saigh P. The development and validation of the Children’s Posttraumatic Stress Disorder Inventory. *International Journal of Special Education* 1989; **4**(1): 75-84.
31. Solomon Z, Benbenishty, R., Neria, Y., Abramowitz, M., Ginzburg, K., & Ohry, A. . Assessment of PTSD: Validation of the revised PTSD Inventory. *Israel Journal of Psychiatry and Related Sciences* 1993; **30**: 110-5.
32. Goodman R, Ford T, Richards H, Gatward R, Meltzer H. The Development and Well-Being Assessment: description and initial validation of an integrated assessment of child and adolescent psychopathology. *The Journal of Child Psychology and Psychiatry and Allied Disciplines* 2000; **41**(5): 645-55.
33. Beck AT, Epstein N, Brown G, Steer RA. An inventory for measuring clinical anxiety: psychometric properties. *Journal of consulting and clinical psychology* 1988; **56**(6): 893.
34. Spitzer RL, Kroenke K, Williams JB, Löwe B. A brief measure for assessing generalized anxiety disorder: the GAD-7. *Archives of internal medicine* 2006; **166**(10): 1092-7.
35. Sheehan DV, Sheehan KH, Shytle RD, et al. Reliability and validity of the mini international neuropsychiatric interview for children and adolescents (MINI-KID). *The Journal of clinical psychiatry* 2010.
36. Reynolds CR, Richmond BO. What I think and feel: A revised measure of children's manifest anxiety. *Journal of abnormal child psychology* 1978; **6**(2): 271-80.
37. Taylor JA. A personality scale of manifest anxiety. *The Journal of abnormal and social psychology* 1953; **48**(2): 285.
38. StataCorp. Stata Statistical Software: Release 11. College Station, Texas: StatCorp LP; 2009.
39. Mickey RM, Greenland S. The impact of confounder selection criteria on effect estimation. *American journal of Epidemiology* 1989; **129**(1): 125-37.
40. Bendel RB, Afifi AA. Comparison of stopping rules in forward “stepwise” regression. *Journal of the American Statistical Association* 1977; **72**(357): 46-53.
41. Charlson FJ, Flaxman A, Ferrari AJ, Vos T, Steel Z, Whiteford HA. Post-traumatic stress disorder and major depression in conflict-affected populations: an epidemiological model and predictor analysis. *Global Mental Health* 2016; **3**.
42. Barendregt JJ. Ersatz Version 1.2. 2012. http://www.epigear.com/index_files/ersatz.html (accessed 14 November 2017.
43. Barendregt JJ. The effect size in uncertainty analysis. *Value in Health* 2010; **13**(4): 388-91.
44. Vos T, Allen C, Arora M, et al. Global, regional, and national incidence, prevalence, and years lived with disability for 310 diseases and injuries, 1990–2015: a systematic analysis for the Global Burden of Disease Study 2015. *The Lancet* 2016; **388**(10053): 1545-602.
45. Whiteford HA, Degenhardt L, Rehm J, et al. Global burden of disease attributable to mental and substance use disorders: findings from the Global Burden of Disease Study 2010. *The Lancet* 2013; **382**(9904): 1575-86.
46. Ferrari AJ, Charlson FJ, Norman RE, et al. Burden of depressive disorders by country, sex, age, and year: findings from the global burden of disease study 2010. *PLoS medicine* 2013; **10**(11): e1001547.
47. Vos T, Abajobir AA, Abate KH, et al. Global, regional, and national incidence, prevalence, and years lived with disability for 328 diseases and injuries for 195 countries, 1990–2016: a systematic analysis for the Global Burden of Disease Study 2016. *The Lancet* 2017; **390**(10100): 1211-59.
